# Real-world safety profile of T-cell engagers: evidence from multi-database analysis with CAR-T comparisons

**DOI:** 10.3389/fimmu.2026.1740144

**Published:** 2026-03-06

**Authors:** Jinman Zhong, Chang Chen, Yunman Xu, Yueping He, Jingwen Luo, Changxiu Zhang, Jiewen Tan, Dan Xiong

**Affiliations:** Department of Hematology, The Eighth Affiliated Hospital, Southern Medical University (The First People’s Hospital of Shunde, Foshan), Foshan, China

**Keywords:** chimeric antigen receptor t-cell therapy, immune effector cell-associated neurotoxicity syndrome, pharmacovigilance, real-world evidence, T-cell engager

## Abstract

**Background:**

T-cell engagers (TCEs), “off-the-shelf” immunotherapies are seeing widespread clinical application, yet their real-world safety profile is not fully defined. This study aimed to characterize the comprehensive adverse event (AE) profile of TCEs, using chimeric antigen receptor T-cell (CAR-T) therapy as a contextual benchmark.

**Methods:**

A pharmacovigilance study was conducted on AE reports for TCE and CAR-T therapies from FAERS and VigiBase. A multi-level analytical framework integrated disproportionality analysis, time-to-onset modeling, occurrence network analysis, and Immune Effector Cell-Associated Neurotoxicity Syndrome (ICANS) analysis to characterize signals, temporal dynamics, and clinical syndromes.

**Results:**

The proportion of fatal outcomes reported with TCEs significantly increased from 14.3% in 2015 to 23.5% in 2025 (P<0.001). Compared to CAR-T, TCEs showed stronger signals for Infection and Tumor Lysis Syndrome (TLS), while CAR-T showed stronger signals for Cytokine Release Syndrome (CRS) and ICANS. TLS and CRS occurred significantly earlier with TCEs. Network analysis quantified the co-occurrence and clinical severity of the CRS-ICANS-infection triad. The TCE class showed profound drug-specific heterogeneity, including severe oral/nail toxicities with talquetamab (oral toxicity ROR = 6066.40) and extramedullary relapse/infiltration with blinatumomab. TCE-associated ICANS revealed a strong overall signal (ROR 197.08), with fatal outcomes reported in 26% of cases, an early-peaking reporting pattern (WSP α = 0.63), and key risk factors including age, indication, target and concurrent CRS.

**Conclusion:**

TCEs are characterized by rapid early CRS/TLS AEs, elevated infection reporting, and target-specific toxicities, while CAR-T exhibits stronger CRS/ICANS signals. These findings support early monitoring and molecule-specific, syndrome-based risk management, advancing precision pharmacovigilance for T-cell redirecting therapies.

## 1 Introduction

T-cell-engaging immunotherapies have marked a pivotal breakthrough in oncology, fundamentally altering the treatment paradigm for hematological malignancies (1, 2). Among these transformative strategies, T-cell engagers (TCEs) are a powerful and versatile therapeutic class (3, 4). These engineered biologic molecules are designed to form a cytolytic bridge between T-cells and tumor cells, redirecting the immune system to eradicate cancer (5). A key distinguishing feature of TCEs is their “off-the-shelf” nature, offering an immediately accessible and logistically simpler alternative to personalized cell therapies (6). This advantage has fueled their rapid clinical expansion, with agents like blinatumomab demonstrating profound efficacy not only in relapsed/refractory settings but also gaining established roles in frontline therapy (7–9), such as measurable residual disease (MRD) clearance in B-cell acute lymphoblastic leukemia where it increases MRD negativity rates from 72% to 93% (10).

The success of TCEs has occurred alongside the parallel development of chimeric antigen receptor T-cell (CAR-T) therapy, which has established itself as the other dominant T-cell redirecting platform in hematological oncology (11). Though both approaches trigger robust T-cell activation but differ: TCEs functioning as “off-the-shelf” biologics versus CAR-T as personalized “living drugs” (12). This distinction creates unique logistical, manufacturing, and accessibility profiles that have positioned these therapies as both complementary and competing options for overlapping patient populations (13). However, their expanding clinical use has outpaced the generation of comparative safety evidence from routine practice. Safety profiles derived from the stringent eligibility criteria of pivotal trials may not fully capture the adverse event landscape in real-world settings, where patient populations are broader, more complex, and often more vulnerable (14, 15).

Current safety evidence is predominantly from single-arm clinical trials, post-marketing observational cohorts, or analyses focused on individual drugs or targets. While these studies have established cytokine release syndrome (CRS) and immune effector cell-associated neurotoxicity syndrome (ICANS) as hallmark toxicities shared by both platforms (16, 17). However, there are significant gaps. A comprehensive characterization of the real-world safety profile across the TCE class is incomplete. The absence of direct, systematic comparisons with CAR-T obscures the unique toxicity profiles, which is essential for optimizing clinical decision-making and patient selection. Research has depended on basic disproportionality analyses, paying little attention to adverse event dynamics such as onset timing, risk evolution, or co-occurrence patterns, which restricts early risk detection and tailored management.

This study was designed to address these knowledge gaps through a comprehensive, multi-database pharmacovigilance investigation into the real-world safety of TCEs. We leveraged two of the world’s largest pharmacovigilance databases, the US FDA Adverse Event Reporting System (FAERS) and WHO’s VigiBase, characterize the full spectrum of TCE-associated adverse events. Given that TCEs and CAR-T therapies share T-cell redirection mechanisms but differ fundamentally in biological properties and delivery contexts, we included CAR-T as a reference framework to contextualize TCE safety signals. We implemented a multi-level analytical framework that integrates advanced signal detection with temporal dynamic modeling and network analysis. By systematically comparing the temporal dynamics, clustering patterns, and target-specific toxicity profiles of TCEs, this study aims to generate evidence-based insights for clinical risk assessment and precision safety management.

## 2 Methods

### 2.1 Data sources and study population

This retrospective pharmacovigilance study was conducted utilizing data from two distinct spontaneous reporting system (SRS) databases: the FAERS and the VigiBase. Adverse events (AEs) were coded in accordance with the Medical Dictionary for Regulatory Activities (MedDRA), version 28.0.

Key adverse events of interest—cytokine release syndrome (CRS), immune effector cell–associated neurotoxicity syndrome (ICANS), tumor lysis syndrome (TLS), and infections—were defined *a priori* using prespecified MedDRA Preferred Term lists. To ensure consistency across databases, the same PT-based definitions were applied in both FAERS and VigiBase, and the two databases were analyzed in parallel to allow independent validation of safety signals.

Data were extracted from the FAERS database for the period spanning the first quarter of 2014 to the second quarter of 2025. To ensure comprehensive case retrieval, drug names, including synonyms and aliases obtained from the FDA Drugs database, were used to search FAERS. The study cohort was limited to reports wherein a TCE or CAR-T therapy was explicitly designated as the primary suspect (PS) drug.

The FAERS dataset was processed using a multi-step protocol aligned with FDA guidelines to ensure data integrity, starting with the removal of technical duplicates by retaining only the most recent version of a report (determined by CASEID, FDA_DT, and PRIMARYID). This was followed by the elimination of content-based duplicates, defined as records with identical values across key demographic and clinical fields. The dataset was then finalized through a cleaning process that excluded invalid reports and removed withdrawn cases from FDA quarterly deletion lists.

To validate and complement the primary analysis, corresponding aggregated data were retrieved from VigiBase up to July 27, 2025. As VigiBase presents results for the active ingredient (often including more than one brand name) and provides cleaned, summary-level data, the extensive deduplication process applied to FAERS was not required. The inclusion criteria were conceptually identical, focusing on reports where TCEs or CAR-T therapies were the suspected agents.

### 2.2 Signal detection

The identification of potential safety signals was achieved through disproportionality analysis. A comprehensive multi-method approach was implemented, incorporating eight distinct statistical algorithms to enhance the robustness of signal detection (18, 19). The employed algorithms included: the Reporting Odds Ratio (ROR) (20), the Proportional Reporting Ratio (PRR) (21), Fisher’s exact test (22), a frequentist Observed-to-Expected (O/E) ratio analysis (23), the Chi-square (*χ*²) test (24), two distinct implementations of the Bayesian Confidence Propagation Neural Network (BCPNN) (25), and the Empirical Bayes Geometric Mean (EBGM) (26). The specific algorithms and their respective thresholds for a positive signal are detailed in **Table 1**. For the primary analysis and data visualization within this study, the ROR was utilized as the principal measure of association strength.

**Table 1:** Signal detection algorithms and thresholds for statistical significance.

Algorithm	Abbreviation	Criteria for positive signal
Reporting Odds Ratio	ROR	ROR_025_ > 1; a ≥ 3
Proportional Reporting Ratio	PRR	PRR_025_ ≥ 1; a ≥ 3
Chi-square Test	*χ²*	*χ*² ≥ 4; PRR ≥ 2; a ≥ 3
Fisher’s Exact Test	–	*P* < 0.05
Observed-to-Expected Ratio	O/E	O/E_025_ > 1
Bayesian Confidence Propagation Neural Network (Normal Approximation)	BCPNN (Norm)	IC_025_ > 0
Bayesian Confidence Propagation Neural Network (Markov Chain Monte Carlo)	BCPNN (MCMC)	IC_025_ > 0
Empirical Bayes Geometric Mean	EBGM	EBGM_05_ ≥ 2

The signal detection strategy was executed across three distinct comparative frameworks: (a) TCEs vs. FAERS Background: Reports involving TCEs were compared against the background of all other drug reports within the FAERS database. (b) CAR-T vs. FAERS Background: A similar comparison was performed for CAR-T therapies against the database background. (c) TCEs vs. CAR-T: A comparative analysis was conducted to evaluate the relative safety profiles of the two therapeutic classes. This analysis was performed independently in both the FAERS and VigiBase databases.

To specifically identify signals that were highly exclusive to a single drug within the TCE cohort, an exploratory metric termed the “Drug-Specificity Ratio” was calculated for each AE by dividing the highest ROR for that event by the second-highest ROR among all other drugs in the cohort. A drug-AE pair was classified as a “drug-specific signal” if it met all of the following criteria: First, standard signals were first identified based on a statistically significant disproportionality (lower bound of the ROR 95% CI > 1) and a minimum threshold of three individual reports (a ≥ 3). Subsequently, to prioritize drug-specific toxicities and distinguish them from class-wide effects, these established signals were filtered using two exploratory criteria: an ROR > 5 and a Drug-Specificity Ratio > 10. An infinite ratio, which occurred if the event was reported for only one drug, was considered to have met the drug-specificity criterion.

### 2.3 Time-to-event analysis

The time-to-onset (TTO) for AEs of special interest was defined as the interval between the drug administration start date (START_DT) and the AE onset date (EVENT_DT). The distribution of TTO was visualized using density plots. The cumulative incidence of AEs was estimated using the Kaplan-Meier method. To further characterize the reporting distribution behavior over time, a Weibull shape parameter (WSP) analysis was also conducted (27).

### 2.4 Co-occurrence and network analysis

To investigate clinical association patterns among significant TCE-related AE signals, a co-occurrence network analysis was performed (28). The analysis included AEs with a Benjamini-Hochberg corrected Fisher’s exact test *P*-value < 0.05. The association strength between co-occurring AE pairs was quantified using the Lift metric. An undirected graph was constructed from pairs with a co-occurrence count of at least 10 and a Lift value > 2. In this network, nodes represent AEs (Preferred Terms), and edges are weighted by the Lift value. The Louvain community detection algorithm (29) was applied to identify densely connected AE clusters, which may represent clinical syndromes. Within each community, weighted eigenvector centrality was calculated as a measure of influence, allowing for the identification of the most prominent hub AEs.

### 2.5 Statistical analysis

The characteristics of the patient cohorts were analyzed using two approaches to ensure both transparency and statistical validity. For broad descriptive visualizations of a single cohort, all reports were included, and missing data were explicitly quantified and displayed as a separate category. For direct statistical comparisons between the TCE and CAR-T cohorts, a complete-case analysis approach was used for each variable. Continuous variables were summarized as median and interquartile range (IQR) and compared using the Wilcoxon rank-sum test. Categorical variables were summarized as frequency and percentage, with percentages based on the total number of non-missing reports for that variable and compared using Pearson’s Chi-squared test or Fisher’s exact test, as appropriate. The Cochran-Armitage test was used to assess for a significant trend in the proportion of fatal outcomes over time.

All statistical computations and data visualizations were executed using R software (Version 4.4.1). For all inferential statistical tests, a *P*-value of less than 0.05, or a corresponding confidence interval excluding the null value, was established as the threshold for statistical significance.

## 3 Results

### 3.1 Descriptive characteristics of T-cell engager AE reports

**Figure 1:**
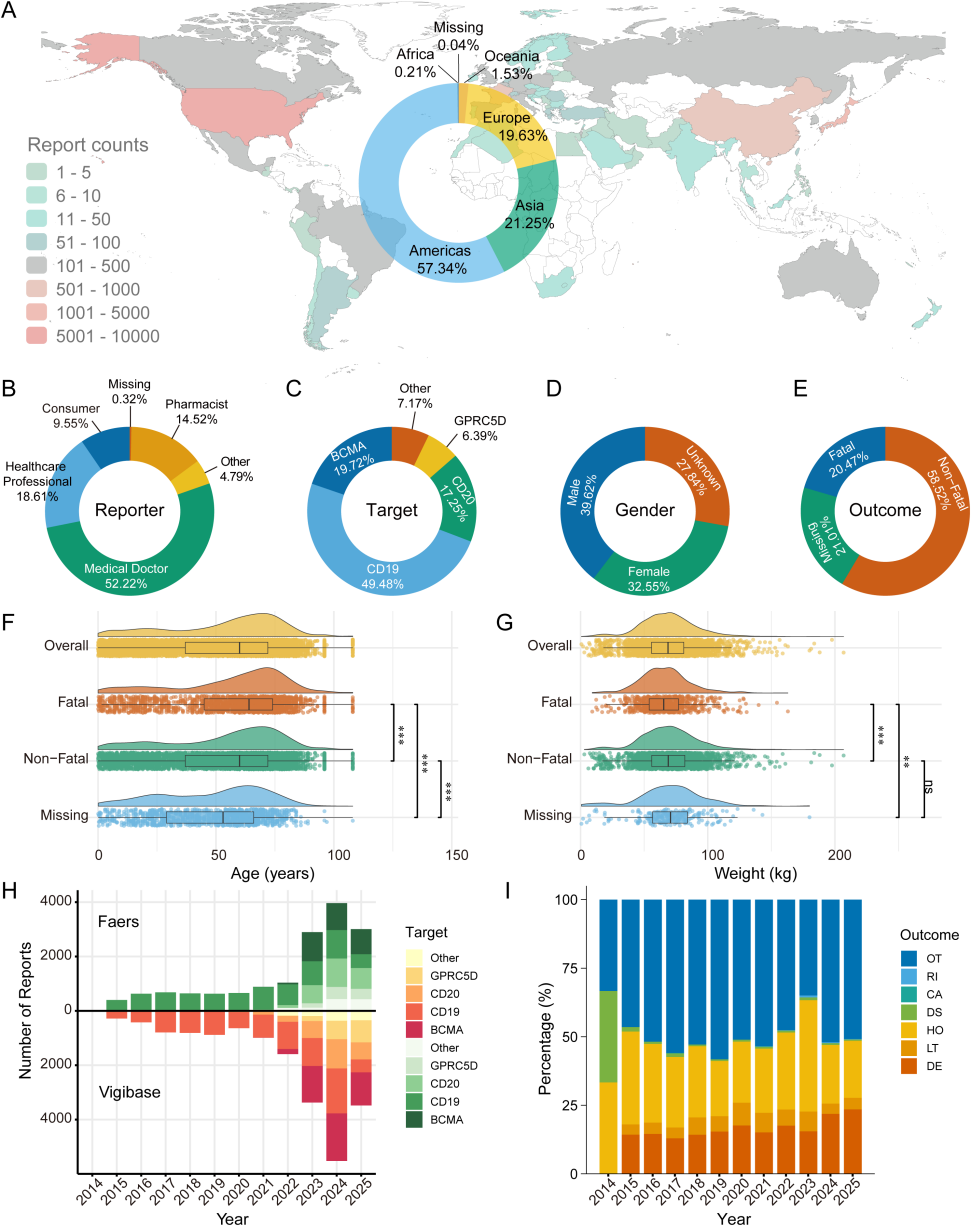
Unless otherwise specified, analyses in this figure are based on the FAERS database. **(A)** Geographic distribution of TCE-associated adverse event reports. The world map is colored by the number of reports per country, and the accompanying donut chart shows the proportional distribution by continent. **(B)** Proportional distribution of report sources, categorized by the professional identity of the reporter. **(C)** Proportional distribution of reports based on the molecular target of the TCE drug. **(D)** Proportional distribution of reports by patient gender. **(E)** Proportional distribution of the most severe outcomes reported. **(F)** Raincloud plot illustrating the age distribution across the overall population and stratified by outcome (fatal, non-fatal, and missing). Statistical comparisons were performed using the Kruskal-Wallis test with Benjamini-Hochberg correction for multiple comparisons. **(G)** Raincloud plot showing the weight distribution across the same population groups as in **(F)**, with statistical analysis performed similarly. **(H)** Bidirectional stacked area chart illustrating the number of reports for TCEs from the FAERS (top) and VigiBase (bottom) databases, categorized by molecular target from 2014 to 2025. FAERS data is current through Q2 2025, and VigiBase data is current through July 27, 2025. **(I)** Stacked bar chart showing the temporal trend in the proportion of different adverse event outcomes. Outcome abbreviations: DE, Death; LT, Life-Threatening; HO, Hospitalization (Initial or Prolonged); DS, Disability; CA, Congenital Anomaly; RI, Required Intervention to Prevent Permanent Impairment/Damage; OT, Other Serious. Significance levels: *** *P* < 0.001; ** *P* < 0.01; ns, *P* ≥ 0.05.

A total of 15,440 AE reports from the FAERS database listing a TCE as the primary suspect drug were identified. The majority of reports originated from the Americas (57.34%), followed by Asia (21.25%) and Europe (19.63%) (**Figure 1A**). Medical doctors (52.22%) were the primary source of reports, followed by healthcare professionals (18.61%) and pharmacists (14.52%) (**Figure 1B**). Among TCE targets, CD19-directed therapies accounted for the largest proportion of reports (49.48%), followed by BCMA (19.72%), CD20 (17.25%), and GPRC5D (6.39%) (**Figure 1C**). Reports were more frequent for male patients (39.62%) than female patients (32.55%), with 27.84% of reports having an unknown gender (**Figure 1D**). Fatal outcomes were reported in 20.47% of cases (**Figure 1E**). Among reports with available data, the median age of the cohort was 60 years (IQR: 37-72), and the median age of patients with fatal outcomes was significantly higher than those with non-fatal outcomes (*P* < 0.001) (**Figure 1F**). Similarly, the median weight was 68.8 kg (IQR: 55.8-81.2), which also differed significantly between outcome groups (*P* < 0.001), with lower weights observed in the fatal outcome group (**Figure 1G**). A marked increase in the number of TCE-associated AE reports was observed from 2014 to 2025 in both FAERS and VigiBase, particularly after 2021 (**Figure 1H**). Concurrently, the proportion of FAERS reports documenting a fatal outcome demonstrated a statistically significant increasing trend over the study period, rising from 14.3% in 2015 to 23.5% in 2025 (Cochran-Armitage test for trend, *P* < 0.001) (**Figure 1I**).

### 3.2 Adverse event profile and co-occurrence patterns of T-cell engagers

**Figure 2:**
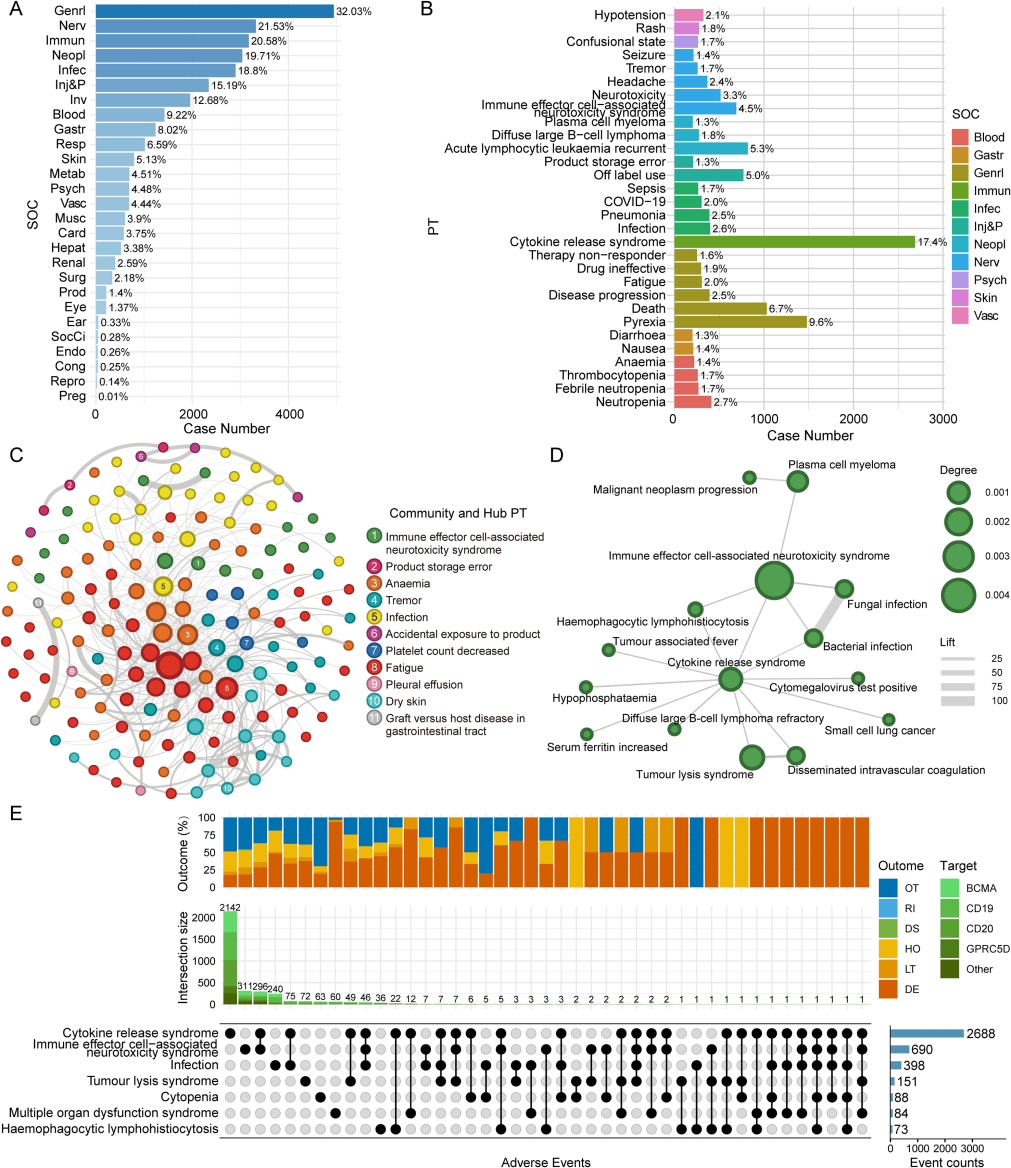
**(A)** Bar chart displaying the percentage and case count of adverse event reports across MedDRA System Organ Classes (SOCs). **(B)** Bar chart showing the 30 most frequently reported Preferred Terms (PTs), with bars colored by their corresponding SOC. **(C)** Co-occurrence network of statistically significant adverse drug reactions (ADRs). Each node represents an ADR, sized by its reporting frequency. Nodes are colored by communities identified via modularity analysis. Edges signify co-occurrence, with their thickness proportional to the lift value (a measure of association strength; higher values indicate stronger co-occurrence). The legend identifies the hub PT for the eleven largest communities, with each community represented by a numbered and colored circle. **(D)** A detailed view of the co-occurrence sub-network for Community 1, centered on the hub PT “immune effector cell-associated neurotoxicity syndrome” (ICANS). Node size is proportional to its degree (number of connections to other events). Edge thickness represents the lift value, indicating the strength of association (thicker edges indicate stronger co-occurrence). **(E)** UpSet plot visualizing the intersections of selected adverse events of interest. The matrix indicates specific combinations of co-reported AEs. The horizontal bar plot on the right shows the total report count for each individual AE. The vertical bar plot above shows the size of each intersection, with bars stacked by drug target. The uppermost stacked bar chart illustrates the proportional distribution of outcomes for each specific intersection. Outcome abbreviations: DE, Death; LT, Life-Threatening; HO, Hospitalization (Initial or Prolonged); DS, Disability; RI, Required Intervention to Prevent Permanent Impairment/Damage; OT, Other Serious. SOC abbreviations: Genrl, General disorders and administration site conditions; Nerv, Nervous system disorders; Neopl, Neoplasms benign, malignant and unspecified (incl cysts and polyps); Blood, Blood and lymphatic system disorders; Inv, Investigations; Gastr, Gastrointestinal disorders; Resp, Respiratory, thoracic and mediastinal disorders; Skin, Skin and subcutaneous tissue disorders; Metab, Metabolism and nutrition disorders; Psych, Psychiatric disorders; Vasc, Vascular disorders; Musc, Musculoskeletal and connective tissue disorders; Card, Cardiac disorders; Hepat, Hepatobiliary disorders; Renal, Renal and urinary disorders; Surg, Surgical and medical procedures; Prod, Product issues; Immun, Immune system disorders; Eye, Eye disorders; SocCi, Social circumstances; Ear, Ear and labyrinth disorders; Endo, Endocrine disorders; Cong, Congenital, familial and genetic disorders; Repro, Reproductive system and breast disorders; Preg, Pregnancy, puerperium and perinatal conditions; Inj&P, Injury, poisoning and procedural complications; Infec, Infections and infestations.

Using FAERS data, the most frequently reported AEs by System Organ Class (SOC) were “general disorders and administration site conditions” (32.03%), “Nervous system disorders” (21.53%), and “immune system disorders” (20.58%) (**Figure 2A**). At the Preferred Term (PT) level, “cytokine release syndrome” was the most common AE, accounting for 17.4% of reports, followed by “pyrexia” (9.6%) and “death” (6.7%) (**Figure 2B**).

The co-occurrence network analysis of FAERS reports partitioned 160 significant AEs into 11 distinct communities with a high modularity score of 0.6672, indicating a robust and clinically meaningful structure (**Figure 2C**; **Supplementary Table 1**). These communities successfully segregated AEs by clinical theme. The most prominent clusters by size included Community 8 (n=46), a group of systemic constitutional symptoms anchored by “fatigue”; Community 3 (n=31), which grouped myelosuppression and its infectious complications around the “anemia” hub; Community 4 (n=14), a cluster of neurological events centered on “tremor”; and Community 10 (n=13), a distinct dermatological and oral toxicity profile with “dry Skin” as a dominant hub. The analysis revealed a critical hyperinflammation cluster in Community 1 (n=15), which centered on ICANS. The internal topology showed that ICANS’s strongest link was to CRS (Lift=2.71). CRS then served as the direct bridge to systemic complications like “tumor lysis syndrome” and “disseminated intravascular coagulation”. In parallel, ICANS demonstrated its own direct, strong connections to “hemophagocytic lymphohistiocytosis” (Lift=2.18) and severe infections within its cluster, such as “bacterial infection” (Lift=6.32) and “fungal infection” (Lift=5.72) (**Figure 2D**).

To dissect these co-occurrence patterns further, an UpSet plot analysis was performed on key AEs of interest in FAERS (**Figure 2E**). This analysis revealed that while CRS occurring alone was the most frequent scenario, the most common pattern was concurrent CRS and ICANS. Importantly, the analysis demonstrated a clear hierarchy of clinical severity: the proportion of fatal and life-threatening outcomes was substantially higher for the CRS-ICANS combination compared to CRS alone. This severity was further amplified when infection was also present, as the triad of CRS, ICANS, and infection was associated with a notably higher proportion of fatal outcomes compared to both CRS alone and the CRS-ICANS combination. This analysis characterizes the safety profile of TCEs, highlighting shared signatures of T-cell activation therapies such as CRS and ICANS. To further contextualize these findings, comparative analysis with CAR-T therapy was performed.

### 3.3 Comparative and drug-specific safety profiles

For comparative analysis using FAERS, 16,212 reports for CAR-T therapies were included. The two cohorts differed significantly in baseline characteristics, with the CAR-T cohort having a higher median weight and a greater proportion of male patients, while the TCE cohort included a larger proportion of elderly patients (≥75 years) (**Table 2**). Against this backdrop, a detailed comparison of AE signals revealed distinct safety profiles between therapeutic classes and individual drugs, reflecting their different modalities as an off-the-shelf biologic versus a personalized living drug. At a class level, disproportionality analysis consistently across both the FAERS and VigiBase databases showed that AEs such as “off label use” and product-related issues were more strongly associated with TCEs, whereas CRS and ICANS were more strongly associated with CAR-T therapies (**Supplementary Figure 1A, B**).

**Table 2:** Characteristics of patients in the T-cell Engager and CAR-T cohorts derived from the FAERS database.

Characteristic	Treatment Cohort		*P* value
	T-cell engagers	CAR-T therapies	
	N = 15,440	N = 16,212	
Age, years*^1^*	60 [37-72]	62 [50-70]	<0.001
Missing	6,819	6,457	
Age group			<0.001
<65	5,070 (58.81%)	5,544 (56.83%)	
≥75	1,567 (18.18%)	1,121 (11.49%)	
65-74	1,984 (23.01%)	3,090 (31.68%)	
Missing	6,819	6,457	
Gender			<0.001
F	5,025 (45.10%)	4,747 (38.55%)	
M	6,117 (54.90%)	7,568 (61.45%)	
Missing	4,298	3,897	
Weight, kg*^1^*	68.8 [55.8-81.2]	75.6 [61.8-89.4]	<0.001
Missing	12,183	10,801	
Weight group			<0.001
<60	1,060 (32.55%)	1,206 (22.29%)	
>80	869 (26.68%)	2,209 (40.82%)	
60-80	1,328 (40.77%)	1,996 (36.89%)	
Missing	12,183	10,801	
Region			<0.001
Africa	32 (0.21%)	1 (0.01%)	
Americas	8,854 (57.37%)	11,839 (73.48%)	
Asia	3,281 (21.26%)	869 (5.39%)	
Europe	3,031 (19.64%)	3,109 (19.30%)	
Oceania	236 (1.53%)	294 (1.82%)	
Missing	6	100	
Reporter			<0.001
Consumer	1,474 (9.58%)	2,671 (17.57%)	
Other	739 (4.80%)	799 (5.26%)	
Health Professional	2,873 (18.67%)	5,314 (34.95%)	
Pharmacist	2,242 (14.57%)	523 (3.44%)	
Physician	8,062 (52.38%)	5,896 (38.78%)	
Missing	50	1,009	
Indication			<0.001
Leukaemia	5,185 (33.58%)	1,547 (9.54%)	
Lymphoma	2,540 (16.45%)	7,598 (46.87%)	
Plasma Cell Neoplasms	2,626 (17.01%)	1,633 (10.07%)	
Solid Tumors	562 (3.64%)	3 (0.02%)	
Unclassified	4,527 (29.32%)	5,431 (33.50%)	
Target			<0.001
BCMA	3,044 (19.72%)	3,970 (24.49%)	
CD19	7,639 (49.48%)	12,242 (75.51%)	
CD20	2,663 (17.25%)	0 (0.00%)	
GPRC5D	987 (6.39%)	0 (0.00%)	
Other	1,107 (7.17%)	0 (0.00%)	

*^1^*Median [Q1-Q3]; n (%).

**Figure 3:**
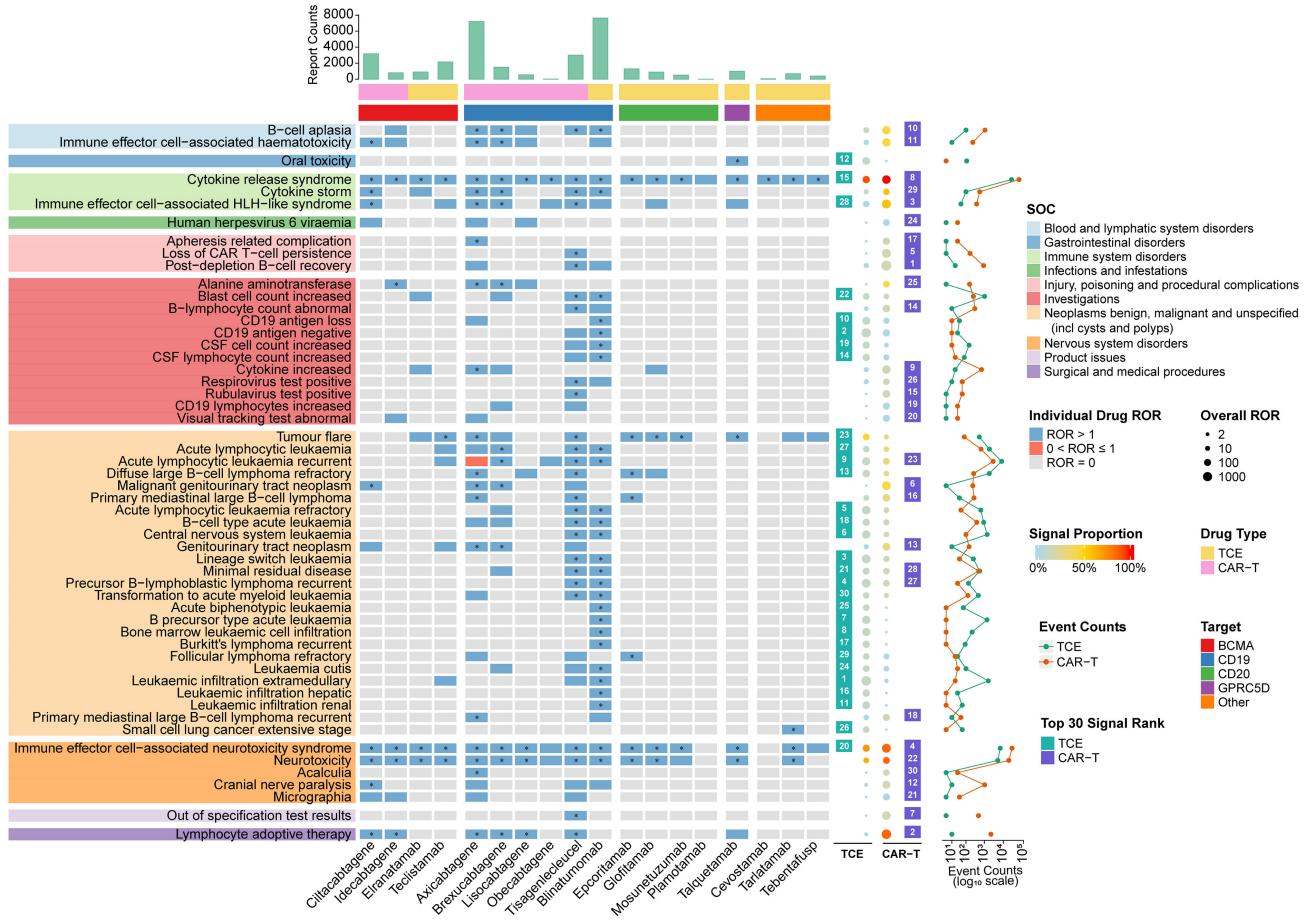
This figure integrates multiple data layers from FAERS to compare the safety profiles of TCEs and CAR-T therapies. Central heatmap (drug-specific signals): Each cell shows the reporting association between a specific drug (column) and an adverse event (row). Blue: indicates a potential safety signal (ROR > 1), meaning the event is reported more frequently than expected. Red: indicates no disproportionate reporting (0 < ROR ≤ 1). Gray: indicates no reports for this drug-event pair (ROR = 0). Asterisk (*): denotes statistical significance (lower 95% CI > 1 and a ≥ 3). Top panels (drug characteristics): Bar chart: displays the total volume of reports for each drug. Color strips: classify drugs by therapy type (yellow for TCE, pink for CAR-T) and molecular target (e.g., CD19, BCMA). Left labels (event categories): Adverse events are grouped by System Organ Class (SOC) (e.g., immune system disorders). Right panels (class-level summary): Rank squares: highlight if the event is among the top 30 signals for TCEs (green) or CAR-T (purple). Bubble plot: summarizes the overall class signal. Bubble size represents the strength of the association (overall ROR); color intensity represents how consistently the signal appears across drugs in the class (signal proportion). Line plot: shows the total number of event reports on a logarithmic scale.

This class-level distinction was further clarified by examining the top-ranking signals for each modality using FAERS (**Figure 3**). Consistent with their shared mechanism, key immune-related toxicities such as CRS and ICANS were reported with high frequency and significant signals in both classes. However, the signals were stronger and more consistent for CAR-T therapies; for example, CRS ranked as the #8 signal for the CAR-T class with a 100% signal proportion across all agents, while also ranking high for the TCE class at #15. Several AEs were primarily associated with CAR-T therapy, reflecting its nature as a cell therapy product. These included “post-depletion B-cell recovery” (CAR-T Rank #1), an on-target effect of profound B-cell depletion.

In contrast, the TCE class displayed a more heterogeneous safety profile with unique signals. “tumor flare” presented as a differential signal between the two modalities. Although reported for both, it emerged as a significantly stronger signal for the TCE class, for which it ranked as a top 30 AEs with a higher overall ROR compared to CAR-T therapies. Notably, beneath these class-level differences, a deeper analysis revealed striking toxicity specificity for individual drugs within the TCE cohort. The relationships between these specific TCE drugs, their molecular targets, and their reported clinical indications are detailed in **Supplementary Figure 2A**. Building on this, a drug-specificity analysis was performed to formally quantify the specificity of signals, identifying 153 AEs that showed high drug-specificity for a single TCE drug (**Supplementary Figure 2B**). This analysis revealed highly specific drug-toxicity clusters. A prime example was a distinct and cluster of toxicities predominantly associated with the GPRC5D-targeted agent, talquetamab. This profile was characterized by profound oral and nail-related AEs, including PTs with exceptionally high signal strength such as “oral toxicity” (ROR = 6066.40, 95% CI 3011.72-12219.33, a=11), “nail toxicity” (ROR = 812.71, 95% CI 441.79-1495.06, a=11), “nail dystrophy” (ROR = 361.17, 95% CI 185.94-701.51, a=9), “anhidrosis” (ROR = 322.42, 95% CI 132.68-783.48, a=5), “onychomadesis” (ROR = 294.38, 95% CI 220.34-393.30, a=49), “salivary hyposecretion” (ROR = 148.19, 95% CI 66.16-331.93, a=6), “skin toxicity” (ROR = 50.62, 95% CI 29.26-87.57, a=13), and “dry mouth” (ROR = 20.88, 95% CI 16.35-26.67, a=69). The analysis also uncovered more complex patterns related to disease evolution under therapeutic pressure. For instance, a unique cluster of AEs describing atypical leukemia recurrence and evolution, including specific events such as “leukemic infiltration extramedullary”, “leukemic infiltration hepatic”, “leukemic infiltration renal”, “central nervous system leukemia”, and “lineage switch leukemia”, was almost exclusively associated with the CD19-targeted TCE, blinatumomab.

### 3.4 Comparative analysis of adverse events of special interest

**Figure 4:**
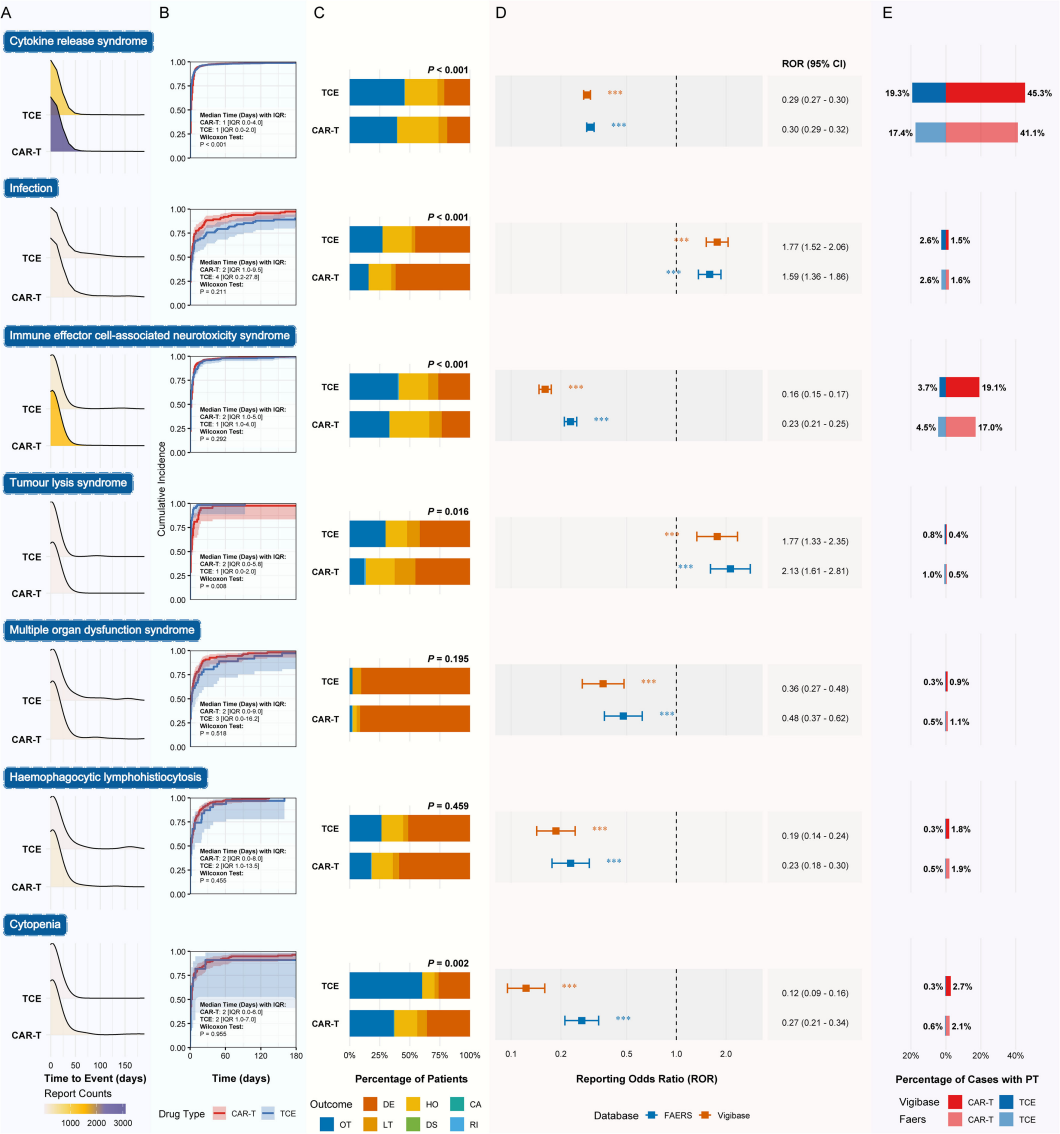
This figure compares key characteristics of seven adverse events of interest between TCE and CAR-T therapies. **(A)** FAERS-derived density plots showing the distribution of time-to-event from drug administration to AE onset. **(B)** Cumulative incidence plots for AE onset over time in the FAERS dataset, with median time-to-onset and interquartile ranges (IQR) provided. P-values are derived from the Wilcoxon test comparing the two groups. **(C)** Stacked bar charts illustrating the proportional distribution of different clinical outcomes for each AE based on FAERS reports. P-values are from Chi-squared tests comparing outcome distributions between TCE and CAR-T. **(D)** Forest plot of Reporting Odds Ratios (RORs) with 95% confidence intervals, comparing TCEs (as the exposure group) versus CAR-T (as the reference group) in both FAERS and VigiBase databases. Significance levels: *** *P* < 0.001. **(E)** Bar charts showing the percentage of cases reporting the specific AE (Preferred Term, PT) within all reports for that drug class, presented separately for the FAERS and VigiBase databases.

To further investigate the differences in safety profiles, a detailed comparative analysis of seven key AEs of interest was conducted between TCEs and CAR-T therapies, focusing on their time-to-onset, clinical outcomes, and reporting disproportionality (**Figure 4**). The analysis of time-to-onset in FAERS revealed significant differences for certain AEs (**Figures 4A, B**). For CRS, although the median onset was 1 day for both therapies, the overall distribution for TCEs (IQR 0.0-2.0) was significantly earlier and more concentrated compared to CAR-T (IQR 0.0-4.0) (*P* < 0.001). A similar trend was observed for tumor lysis syndrome (TLS), where the onset for TCEs (Median 1 day, IQR 0.0-2.0) was also significantly earlier than for CAR-T (Median 2 days, IQR 0.0-5.8) (*P* = 0.008). In contrast, for ICANS, infection, multiple organ dysfunction syndrome, hemophagocytic lymphohistiocytosis, and cytopenia, no statistically significant differences in the time-to-onset distributions were observed between the two drug classes.

Furthermore, the proportional distribution of clinical outcomes in FAERS differed significantly between the two classes for CRS (*P* < 0.001), Infection (*P* < 0.001), ICANS (*P* < 0.001), TLS (*P* = 0.016), and Cytopenia (*P* = 0.002) (**Figure 4C**). No significant difference in the overall outcome composition was found for Multiple organ dysfunction syndrome or hemophagocytic lymphohistiocytosis. Disproportionality analysis using both FAERS and VigiBase revealed a clear divergence in the safety profiles. AEs such as CRS, ICANS, cytopenia, multiple organ dysfunction syndrome, and hemophagocytic lymphohistiocytosis were all disproportionately reported with CAR-T therapies, as indicated by an ROR with a 95% confidence interval upper bound significantly below 1 in both databases (**Figure 4D**). This was further supported by their substantially higher reporting frequencies in the CAR-T cohort (**Figure 4E**). Conversely, infection and tumor lysis syndrome were disproportionately reported with TCEs, exhibiting significant signals with the 95% CI lower bound of the ROR exceeding 1 in both databases. This was further supported by their higher reporting frequencies in the TCE cohort. A breakdown of these signals by molecular target in FAERS further nuanced the comparison; while signals for CRS and ICANS were consistently robust across all CAR-T targets, they varied within the TCE class, with BCMA and GPRC5D targets also showing notable signals. Conversely, the signal for infection was more pronounced across the majority of TCE targets (**Supplementary Figure 1C**). In summary, this focused analysis reveals that for these key shared toxicities, the two technological platforms present distinct reporting dynamics in real-world settings.

### 3.5 In-depth analysis of T-cell engager-associated ICANS

**Figure 5:**
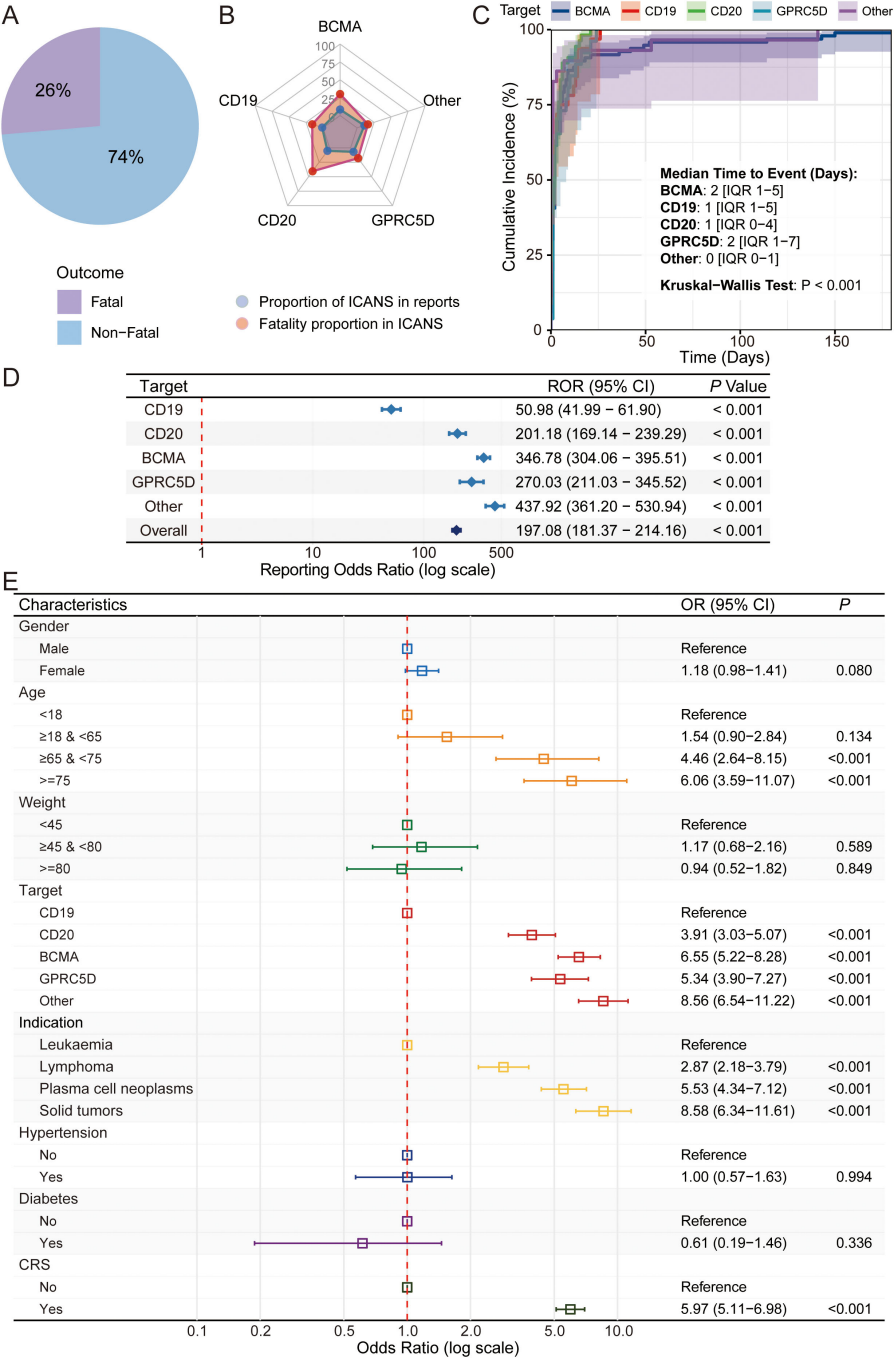
**(A)** Pie chart illustrating the proportion of fatal versus non-fatal outcomes in TCE-associated ICANS reports. **(B)** Radar chart comparing the proportion of ICANS reports (blue) and the fatality proportion within ICANS cases (red) across different TCE molecular target classes. **(C)** Cumulative incidence plots showing the time-to-onset of ICANS, stratified by TCE molecular target. Median time-to-event with interquartile range (IQR) is provided, and the Kruskal-Wallis test was used for group comparison. **(D)** Forest plot of Reporting Odds Ratios (RORs) on a log scale, displaying the signal strength for ICANS associated with the overall TCE class and with each molecular target subclass. **(E)** Forest plot summarizing the results of a univariate analysis of potential risk factors for the occurrence of ICANS. Odds Ratios (ORs) with 95% confidence intervals are shown for various patient and treatment characteristics.

The preceding analyses identified ICANS as a key differentiator between the two advanced T-cell therapies. Given its clinical severity and the observed signal differences, a dedicated in-depth analysis of TCE-associated ICANS was conducted using FAERS data to fully characterize its clinical features, target-related differences, and risk factors. This analysis revealed that 26% of reported ICANS cases had a fatal outcome (**Figure 5A**). Stratified by target, Other-directed TCEs had the highest proportion of ICANS reports, while CD20-directed TCEs had the highest case fatality rate among ICANS reports (**Figure 5B**). The median time to ICANS onset was short across all targets, ranging from 0 to 2 days, with a statistically significant difference observed between the groups (Kruskal-Wallis test, *P* < 0.001) (**Figure 5C**). Strong ICANS signals were detected for the overall TCE class (ROR 197.08, 95% CI 181.37-214.16) and for all TCE targets (**Figure 5D**). WSP analysis of FAERS data revealed distinct time-to-event distribution patterns across different molecular targets (**Supplementary Table 2**). Notably, the analysis for all TCEs combined showed a strong signal for an early-peaking event pattern, with an overall shape parameter (α) significantly below 1 (α = 0.63, 95% CI 0.57-0.69). This early-onset pattern was also observed for BCMA-targeted agents (α = 0.57, 95% CI 0.49-0.65) and the Other-target category (α = 0.52, 95% CI 0.36-0.75), indicating that ICANS events were most frequently reported shortly after treatment initiation. Conversely, for CD19, CD20, and GPRC5D targets, the shape parameters could not be statistically distinguished from 1.0, as their respective 95% confidence intervals all included 1.0, suggesting a relatively constant event reporting rate over time. Univariate analysis using FAERS identified several potential risk factors for the occurrence of ICANS (**Figure 5E**). Compared to their respective reference groups, the risk of ICANS was significantly higher for patients of older age (≥65 years), those with underlying diagnoses of lymphoma, plasma cell neoplasms, or solid tumors (versus leukemia), those with CD20, BCMA, GPRC5D, or Other targets (versus CD19), and those with concurrent CRS (all *P* < 0.001). For fatal outcomes among patients with ICANS, a weight ≥80 kg was protective (*P* = 0.009), while the risk of death was significantly higher for patients with CD20 or BCMA targets (versus CD19), those with underlying diagnoses of lymphoma or plasma cell neoplasms (versus leukemia), and those with concurrent CRS (all *P* < 0.01) (**Supplementary Figure 3**).

## 4 Discussion

This pharmacovigilance study, based on two large global databases, characterizes the reporting patterns of TCEs and contextualizes them against CAR-T therapies as a reference framework. These differences are rooted in their distinct nature as “off-the-shelf” biologics versus personalized “living drugs”. We identified CRS, ICANS, and infection as the central toxicities of TCEs. By detailing their unique onset patterns, co-occurring AEs, and drug-specific risks, this work offers a practical framework for managing these advanced immunotherapies.

Our analysis revealed an increasing proportion of fatal outcomes in TCE reports, rising from 14.3% in 2015 to 23.5% in 2025, paralleling the surge in overall AE reporting after 2021. This reporting trend warrants careful interpretation. Without exposure denominators, we cannot determine whether this reflects a true increase in real-world mortality or changes in reporting patterns. Alternative explanations include expansion to more refractory disease settings as TCEs received broader regulatory approvals, shifts in product mix as new agents entered the market, increased reporting awareness among clinicians, or changes in reporting completeness over time. The observed trend may also reflect that TCEs are increasingly used in frailer, more heavily pretreated patients who would not have qualified for pivotal registration trials (30, 31). The predominance of reports from the Americas likely reflects regional differences in drug accessibility and reporting system maturity, highlighting the need to account for heterogeneity in global data interpretation. Taken together, these findings show an urgent need for evidence-based risk models tailored to real-world patients, moving beyond trial criteria to address the rise in fatal toxicities.

Our AE network analysis shows how toxicities cluster together, which may guide clinicians to monitor and manage them as groups. CRS was the most frequently reported AE in TCEs, affecting 17.4% of reports. Our AE network analysis identified ICANS as a central node with strong connections to CRS. The interplay between CRS and ICANS is well-documented in the cellular therapy literature (32), with CRS-associated systemic inflammation proposed as an initiating factor in the neuroinflammatory cascade (33, 34). The pathophysiology is thought to involve systemic inflammation from CRS leading to widespread endothelial activation, which in turn compromises the integrity of the blood-brain barrier (35), allowing cytokines and immune cells to enter the central nervous system and trigger a distinct, localized neuroinflammatory cascade that manifests as ICANS (36, 37). In our network, ICANS also demonstrated connections to extreme inflammation patterns such as hemophagocytic lymphohistiocytosis, representing the peak of uncontrolled immune activation (38–40). Most critically, ICANS also links to severe bacterial and fungal infections. The UpSet plot analysis quantified this severity hierarchy: reports documenting CRS, ICANS, and infection together were associated with notably higher proportions of fatal outcomes compared to CRS alone or CRS with ICANS. The proposed mechanisms include immune dysregulation from cytokine release and immunosuppressive therapies such as tocilizumab and corticosteroids, which may heighten infection susceptibility (41–43). Notably, our finding of disproportionately higher infection signals with TCEs is corroborated by recent clinical trial evidence. A meta-analysis of 25 prospective trials demonstrated significantly higher per-patient-month infection rates with bispecific antibodies compared to CAR-T, particularly for severe infections (44), underscoring the importance of vigilant infection monitoring in TCE recipients on extended therapy.

Understanding the temporal dynamics of these toxicities is key to early intervention. Our analysis shows that TCEs and CAR-T therapies have different schedules for their AEs based on how they work. TCEs act fast, sparking rapid T-cell activation that starts inflammation and cell damage within the first few days of treatment (5). Our findings confirm that CRS and TLS show up much earlier with TCEs. A WSP analysis of TCEs combined revealed an early-peaking risk for ICANS, with a shape parameter (α = 0.63, 95% CI 0.57-0.69) well below 1, showing the risk drops over time. Meanwhile, CAR-T cells, as “living drugs,” take time to grow in the body, with T-cell and inflammation levels rising days to weeks after infusion (45). This slower but longer-lasting effect brings more intense and prolonged CRS and ICANS in CAR-T patients. Interestingly, while CRS and TLS hit sooner with TCEs, later issues like ICANS and severe infections emerge at similar times in both treatments. This suggests that once inflammation takes hold, the timing of these later complications aligns across therapies. To catch these clusters early, TCE patients require intensive monitoring focused on the early treatment period, whereas the window for CAR-T patients must be extended and shifted to a later period.

We identified profound drug- and target-level heterogeneity, supporting a shift toward molecule-specific risk assessment and management. For instance, the distinctive oral, skin, and nail toxicities associated with the GPRC5D-targeted talquetamab stand out, stemming from a direct on-target, off-tumor effect because GPRC5D appears in the keratinized tissues of the skin, tongue, and nail beds (46, 47). With the CD19-directed blinatumomab, unique signals of extramedullary relapse/infiltration and lineage switch are observed. These issues aren’t separate but connect through intense immune pressure, as our AE co-occurrence network analysis groups extramedullary leukemic infiltration events like “central nervous system leukemia” and “extramedullary leukemic infiltration” together, showing they often occur concurrently. Blinatumomab exerts potent CD19-targeted pressure that, while highly effective in the bone marrow, drives immune escape by selecting for CD19-negative clones or inducing lineage switch, leading to either extramedullary relapse or lineage-switched relapse (48–51). The shared underlying mechanisms for both extramedullary relapse and lineage switch include the “sanctuary site” effect, where drugs may have insufficient penetration or immune surveillance is limited, such as in the central nervous system or testes, and the enhanced clonal heterogeneity under CD19-directed pressure. This allows lineage-switched or antigen-loss variants to preferentially establish and proliferate in these extramedullary sites, effectively evading the targeted therapy. Indeed, extramedullary disease and relapse are not unique to blinatumomab, similar challenges of extramedullary progression have been observed with other TCE targeting BCMA in multiple myeloma (52, 53). This suggests a broader challenge in TCEs against uneven drug reach and immune evasion across different tissues. Therefore, clinicians should closely monitor for extramedullary disease and consider combining TCEs with drugs that have better penetration. This also highlights the need for crafting drug-specific treatment plans and exploring novel combination therapies to more effectively tackle these “sanctuary sites”.

We leveraged two global pharmacovigilance databases and applied eight orthogonal signal-detection algorithms, enhancing robustness over single-method analyses. The analysis of time-to-onset distributions and network-based community detection moved beyond simple disproportionality to reveal temporal dynamics and clinically recognizable AE patterns. However, several limitations should be acknowledged. Spontaneous reporting data are subject to under-reporting and reporting biases and lack systematic capture of key covariates such as tumor burden and performance status. A substantial proportion of reports had missing demographic data, which may introduce selection bias. Additionally, FAERS lacks standardized severity grading; MedDRA Preferred Terms classify adverse events by type rather than clinical severity, precluding stratification by CTCAE grade or differentiation between mild and severe infections. Our analysis quantifies reporting-based associations and cannot establish causality or true incidence rates. The co-occurrence network is internally derived and requires external validation. Additionally, TCEs and CAR-T therapies are administered in distinct clinical contexts, with differences in patient selection, treatment settings, and monitoring intensity. Direct comparisons should therefore be interpreted as contextual benchmarking rather than head-to-head comparisons. All risk factor analyses are univariate and may be subject to residual confounding. Time-to-event analyses rely on reported dates that may be imprecise. Future research should prioritize prospective observational studies to estimate the true incidence and severity of these toxicities and to disentangle the independent contributions of potential risk factors. There is an urgent need for mechanistic studies to better understand the biological drivers of ICANS for each therapeutic modality, and for the development of predictive biomarkers for severe CRS and the lethal CRS–ICANS–infection triad. Finally, head-to-head clinical trials comparing TCEs and CAR-T therapies in carefully defined patient subgroups are necessary to guide optimal therapy selection.

## 5 Conclusion

This large-scale analysis demonstrates that TCEs and CAR-T therapies have fundamentally different safety profiles, despite sharing a common therapeutic principle. For CAR-T therapies, the risk profile is concentrated in the intensity of CRS and ICANS. While TCEs also present risks of CRS and ICANS, their safety profile is uniquely defined by faster onset of events such as TLS, elevated signals for infections, and unique, target-specific “on-target, off-tumor” toxicities. These findings argue for a critical shift in clinical practice: moving beyond class-level warnings toward a more granular, molecule-specific approach to risk management. Achieving this objective requires the development of proactive, syndrome-specific care pathways tailored to each therapy’s distinct profile to optimize patient safety and ensure better clinical outcomes.

## Data Availability

The original contributions presented in the study are included in the article/**Supplementary Material**. Further inquiries can be directed to the corresponding author.
