# Supplementary material for: Real-world safety profile of T-cell engagers: evidence from multi-database analysis with CAR-T comparisons
Source: Front Immunol. 2026 Mar 6;17:1740144. doi: 10.3389/fimmu.2026.1740144 (PMC13002793; doi:10.3389/fimmu.2026.1740144)
Supplement: Supplementary file 1 [file DataSheet1.docx]

**Figure Legends**

**
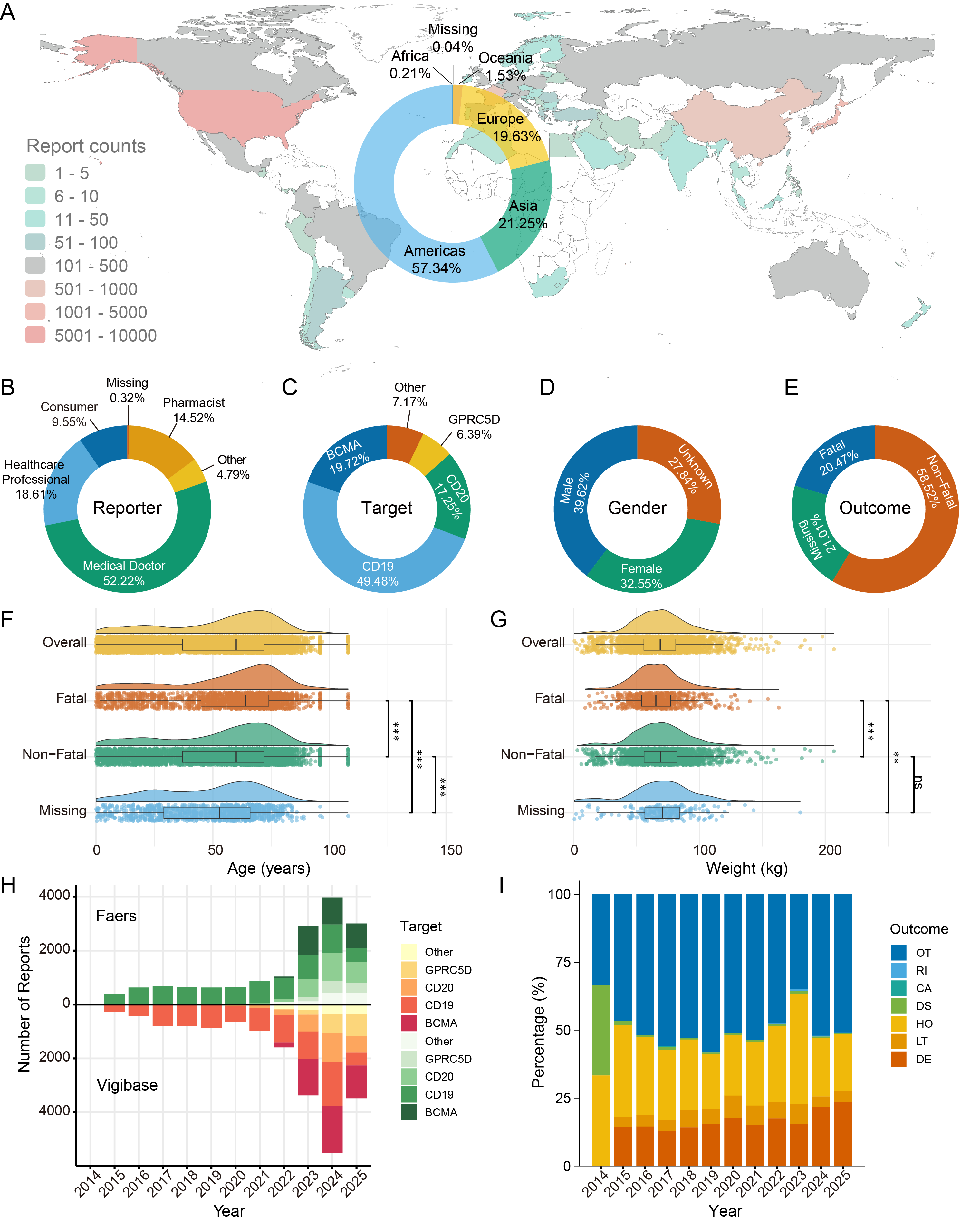
**

**Figure 1. Descriptive Analysis of Adverse Event Reports Associated with T-cell Engagers (TCEs).**

Unless otherwise specified, analyses in this figure are based on the FAERS database. (A) Geographic distribution of TCE-associated adverse event reports. The world map is colored by the number of reports per country, and the accompanying donut chart shows the proportional distribution by continent. (B) Proportional distribution of report sources, categorized by the professional identity of the reporter. (C) Proportional distribution of reports based on the molecular target of the TCE drug. (D) Proportional distribution of reports by patient gender. (E) Proportional distribution of the most severe outcomes reported. (F) Raincloud plot illustrating the age distribution across the overall population and stratified by outcome (fatal, non-fatal, and missing). Statistical comparisons were performed using the Kruskal-Wallis test with Benjamini-Hochberg correction for multiple comparisons. (G) Raincloud plot showing the weight distribution across the same population groups as in (F), with statistical analysis performed similarly. (H) Bidirectional stacked area chart illustrating the number of reports for TCEs from the FAERS (top) and VigiBase (bottom) databases, categorized by molecular target from 2014 to 2025. FAERS data is current through Q2 2025, and VigiBase data is current through July 27, 2025. (I) Stacked bar chart showing the temporal trend in the proportion of different adverse event outcomes. Outcome abbreviations: DE, Death; LT, Life-Threatening; HO, Hospitalization (Initial or Prolonged); DS, Disability; CA, Congenital Anomaly; RI, Required Intervention to Prevent Permanent Impairment/Damage; OT, Other Serious. Significance levels: *** *P* < 0.001; ** *P* < 0.01; ns, *P* ≥ 0.05.


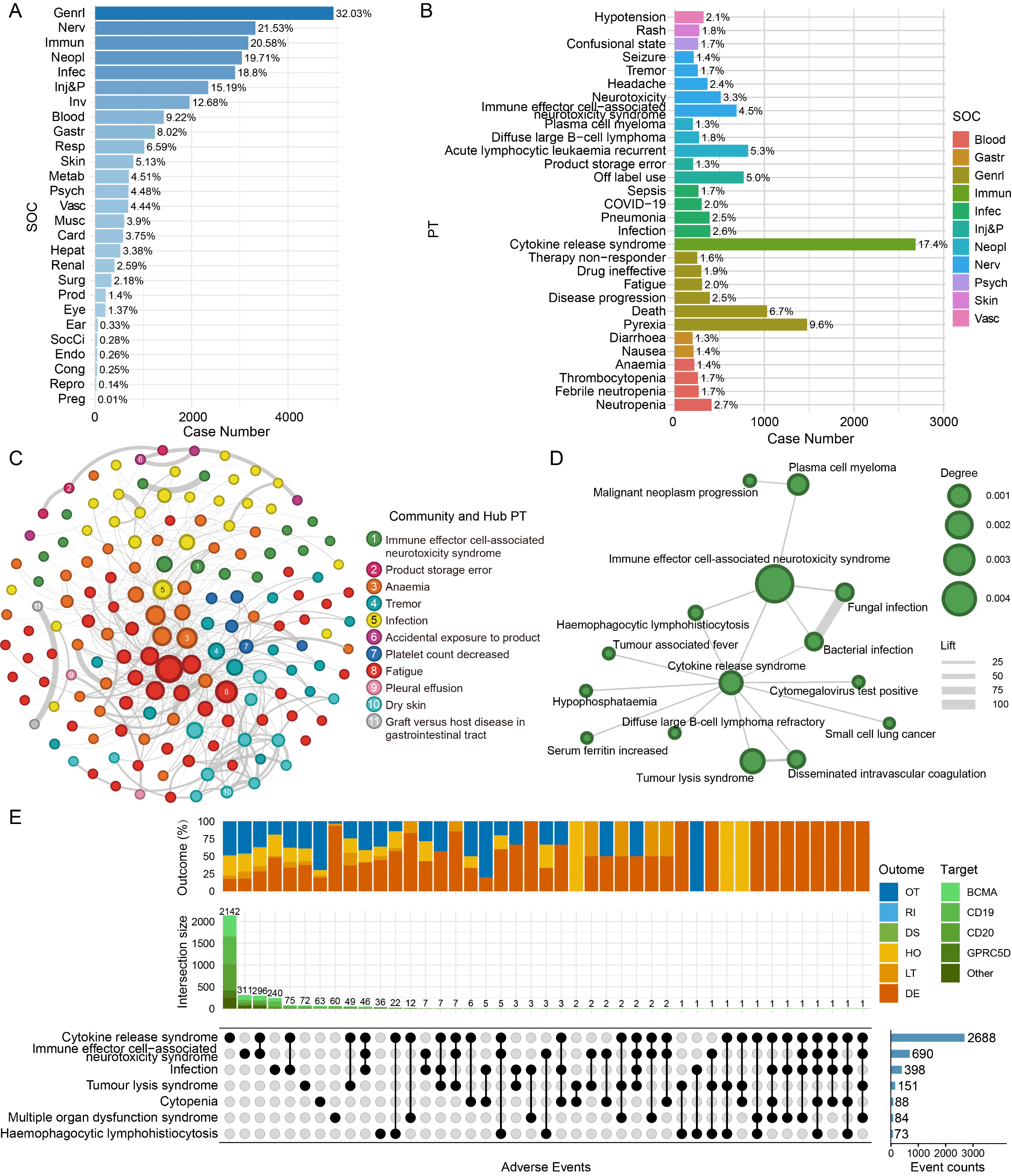


**Figure 2. Characterization and Co-occurrence Patterns of Adverse Events with T-cell Engagers (TCEs) in the FAERS Database.**

(A) Bar chart displaying the percentage and case count of adverse event reports across MedDRA System Organ Classes (SOCs). (B) Bar chart showing the 30 most frequently reported Preferred Terms (PTs), with bars colored by their corresponding SOC. (C) Co-occurrence network of statistically significant adverse drug reactions (ADRs). Each node represents an ADR, sized by its reporting frequency. Nodes are colored by communities identified via modularity analysis. Edges signify co-occurrence, with their thickness proportional to the lift value (a measure of association strength; higher values indicate stronger co-occurrence). The legend identifies the hub PT for the eleven largest communities, with each community represented by a numbered and colored circle. (D) A detailed view of the co-occurrence sub-network for Community 1, centered on the hub PT “immune effector cell-associated neurotoxicity syndrome” (ICANS). Node size is proportional to its degree (number of connections to other events). Edge thickness represents the lift value, indicating the strength of association (thicker edges indicate stronger co-occurrence). (E) UpSet plot visualizing the intersections of selected adverse events of interest. The matrix indicates specific combinations of co-reported AEs. The horizontal bar plot on the right shows the total report count for each individual AE. The vertical bar plot above shows the size of each intersection, with bars stacked by drug target. The uppermost stacked bar chart illustrates the proportional distribution of outcomes for each specific intersection. Outcome abbreviations: DE, Death; LT, Life-Threatening; HO, Hospitalization (Initial or Prolonged); DS, Disability; RI, Required Intervention to Prevent Permanent Impairment/Damage; OT, Other Serious. SOC abbreviations: Genrl, General disorders and administration site conditions; Nerv, Nervous system disorders; Neopl, Neoplasms benign, malignant and unspecified (incl cysts and polyps); Blood, Blood and lymphatic system disorders; Inv, Investigations; Gastr, Gastrointestinal disorders; Resp, Respiratory, thoracic and mediastinal disorders; Skin, Skin and subcutaneous tissue disorders; Metab, Metabolism and nutrition disorders; Psych, Psychiatric disorders; Vasc, Vascular disorders; Musc, Musculoskeletal and connective tissue disorders; Card, Cardiac disorders; Hepat, Hepatobiliary disorders; Renal, Renal and urinary disorders; Surg, Surgical and medical procedures; Prod, Product issues; Immun, Immune system disorders; Eye, Eye disorders; SocCi, Social circumstances; Ear, Ear and labyrinth disorders; Endo, Endocrine disorders; Cong, Congenital, familial and genetic disorders; Repro, Reproductive system and breast disorders; Preg, Pregnancy, puerperium and perinatal conditions; Inj&P, Injury, poisoning and procedural complications; Infec, Infections and infestations.


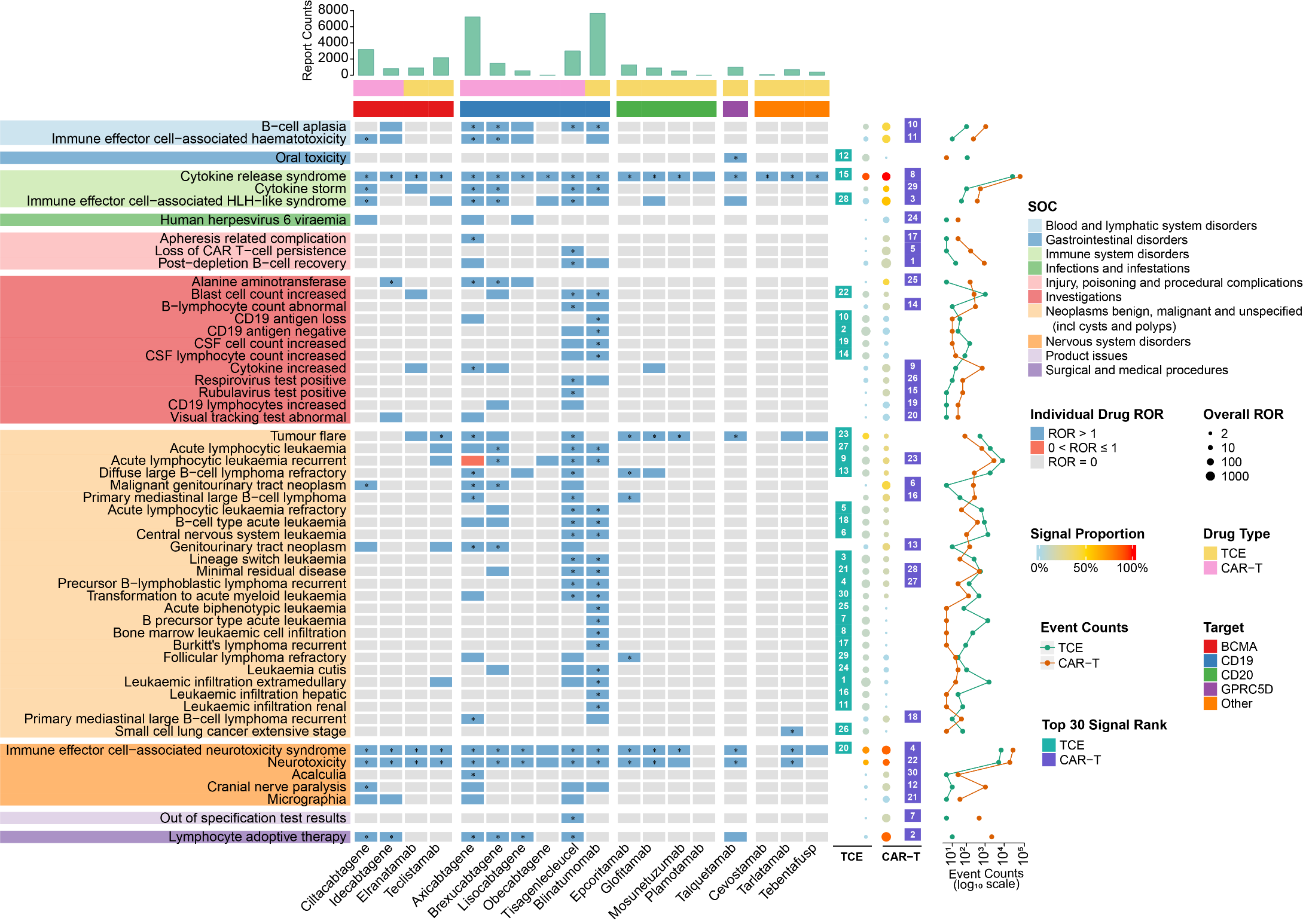


**Figure 3. Comparative Safety Signal Heatmap of T-cell Engagers and CAR-T Therapies**.

This figure integrates multiple data layers from FAERS to compare the safety profiles of TCEs and CAR-T therapies. **Central heatmap (drug-specific signals):** Each cell shows the reporting association between a specific drug (column) and an adverse event (row). Blue: indicates a potential safety signal (ROR > 1), meaning the event is reported more frequently than expected. Red: indicates no disproportionate reporting (0 < ROR ≤ 1). Gray: indicates no reports for this drug-event pair (ROR = 0). Asterisk (*): denotes statistical significance (lower 95% CI > 1 and a ≥ 3). **Top panels (drug characteristics):** Bar chart: displays the total volume of reports for each drug. Color strips: classify drugs by therapy type (yellow for TCE, pink for CAR-T) and molecular target (e.g., CD19, BCMA). Left labels (event categories): Adverse events are grouped by System Organ Class (SOC) (e.g., immune system disorders). **Right panels (class-level summary):** Rank squares: highlight if the event is among the top 30 signals for TCEs (green) or CAR-T (purple). Bubble plot: summarizes the overall class signal. Bubble size represents the strength of the association (overall ROR); color intensity represents how consistently the signal appears across drugs in the class (signal proportion). Line plot: shows the total number of event reports on a logarithmic scale.


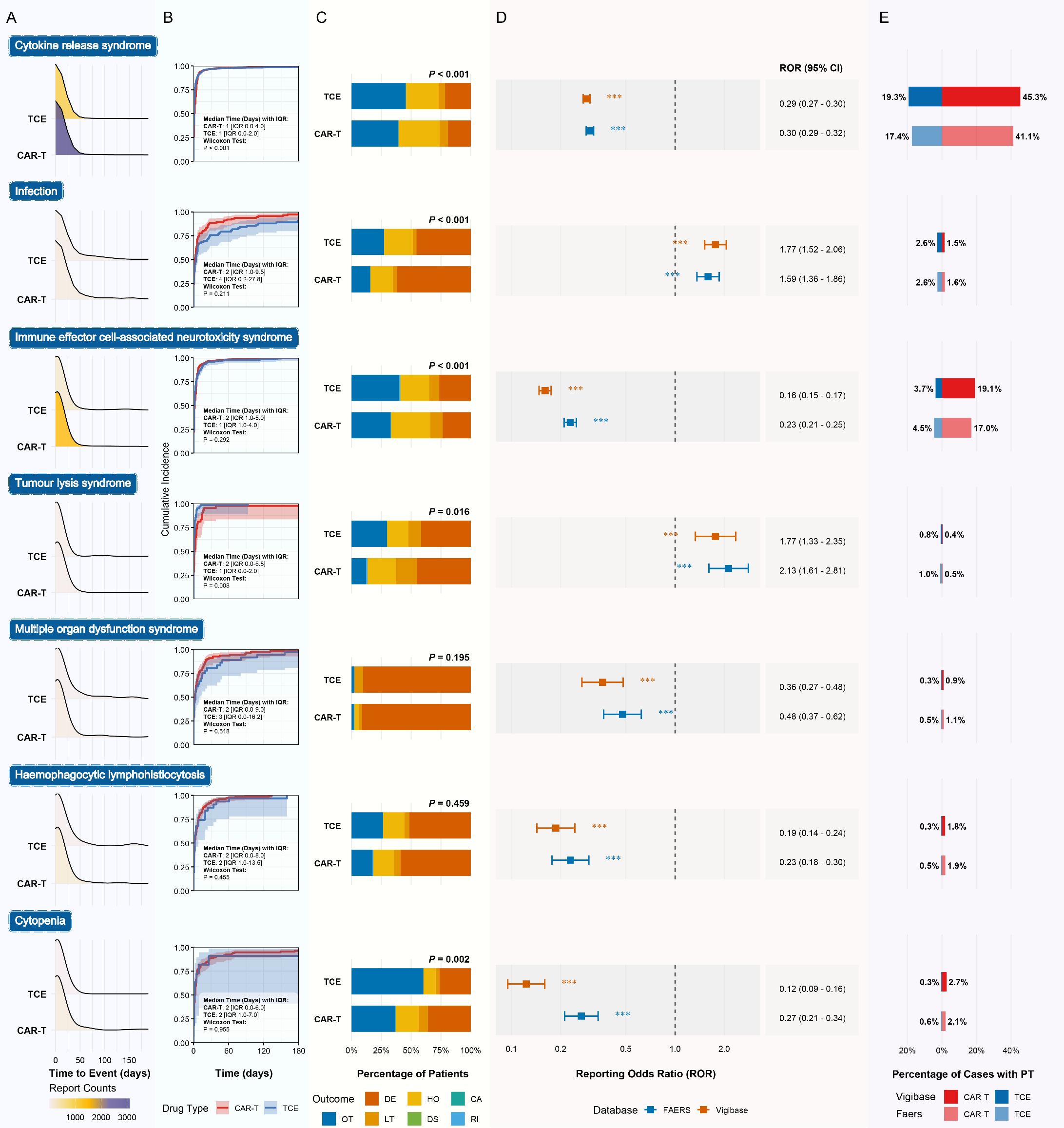


**Figure 4. Comparative Analysis of Selected Adverse Events Between T-cell Engagers (TCEs) and CAR-T Therapies.**

This figure compares key characteristics of seven adverse events of interest between TCE and CAR-T therapies. (A) FAERS-derived density plots showing the distribution of time-to-event from drug administration to AE onset. (B) Cumulative incidence plots for AE onset over time in the FAERS dataset, with median time-to-onset and interquartile ranges (IQR) provided. P-values are derived from the Wilcoxon test comparing the two groups. (C) Stacked bar charts illustrating the proportional distribution of different clinical outcomes for each AE based on FAERS reports. *P*-values are from Chi-squared tests comparing outcome distributions between TCE and CAR-T. (D) Forest plot of Reporting Odds Ratios (RORs) with 95% confidence intervals, comparing TCEs (as the exposure group) versus CAR-T (as the reference group) in both FAERS and VigiBase databases. Significance levels: *** *P* < 0.001. (E) Bar charts showing the percentage of cases reporting the specific AE (Preferred Term, PT) within all reports for that drug class, presented separately for the FAERS and VigiBase databases.


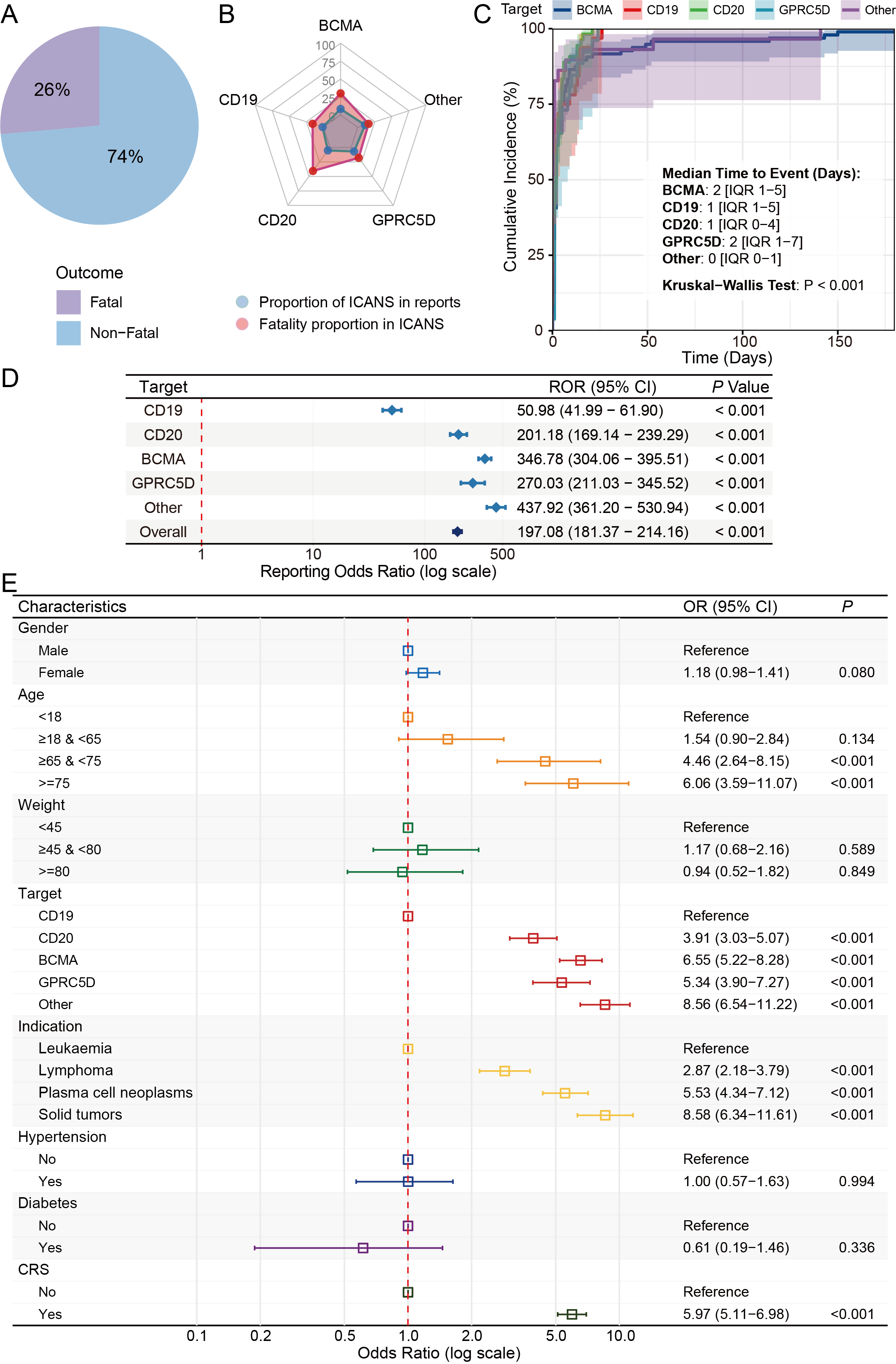


**Figure 5. In-depth Analysis of Immune Effector Cell-Associated Neurotoxicity Syndrome (ICANS) Associated with T-cell Engagers (TCEs) based on FAERS data.**

(A) Pie chart illustrating the proportion of fatal versus non-fatal outcomes in TCE-associated ICANS reports. (B) Radar chart comparing the proportion of ICANS reports (blue) and the fatality proportion within ICANS cases (red) across different TCE molecular target classes. (C) Cumulative incidence plots showing the time-to-onset of ICANS, stratified by TCE molecular target. Median time-to-event with interquartile range (IQR) is provided, and the Kruskal-Wallis test was used for group comparison. (D) Forest plot of Reporting Odds Ratios (RORs) on a log scale, displaying the signal strength for ICANS associated with the overall TCE class and with each molecular target subclass. (E) Forest plot summarizing the results of a univariate analysis of potential risk factors for the occurrence of ICANS. Odds Ratios (ORs) with 95% confidence intervals are shown for various patient and treatment characteristics.

**Supplementary Figure Legends**

**
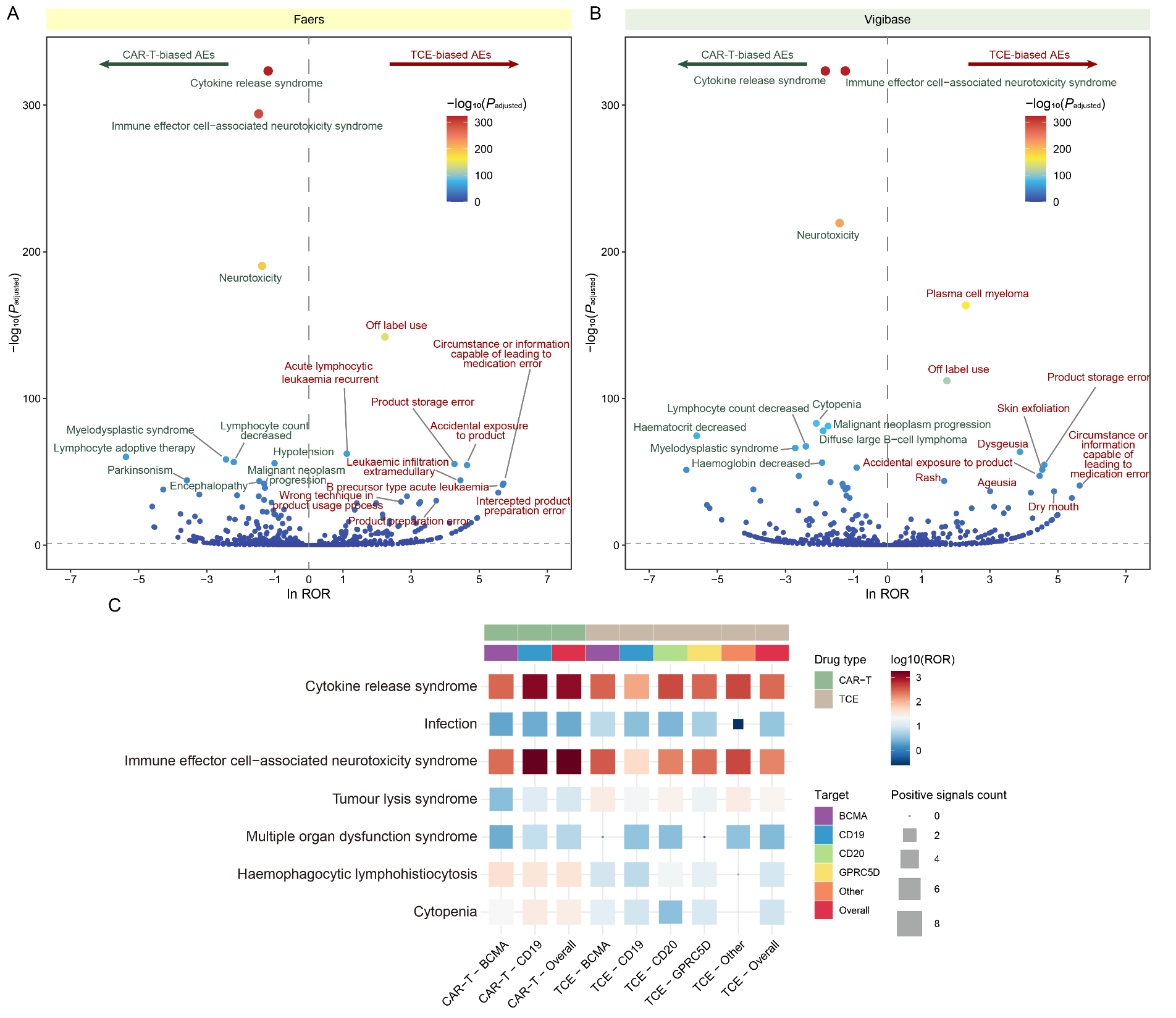
**

**Figure S1. Comparative Disproportionality Analysis of T-cell Engagers (TCEs) versus CAR-T Therapies.**

(A, B) Volcano plots comparing adverse events (AEs) reported for TCEs versus CAR-T therapies in the (A) FAERS and (B) VigiBase databases. The x-axis represents the log-transformed Reporting Odds Ratio (ln ROR), and the y-axis represents statistical significance as the negative log10 of the Benjamini-Hochberg adjusted P-value. The plots identify the top 10 most statistically significant adverse events disproportionately associated with each drug class: those more strongly associated with TCEs (ln ROR > 0) and those more strongly associated with CAR-T therapies (ln ROR < 0). (C) Heatmap displaying the pharmacovigilance signals derived from FAERS for selected AEs across different drug classes and their molecular targets. The color of each square represents the ROR value, while the size of the square is proportional to the number of positive signals detected out of eight different signal detection methods.


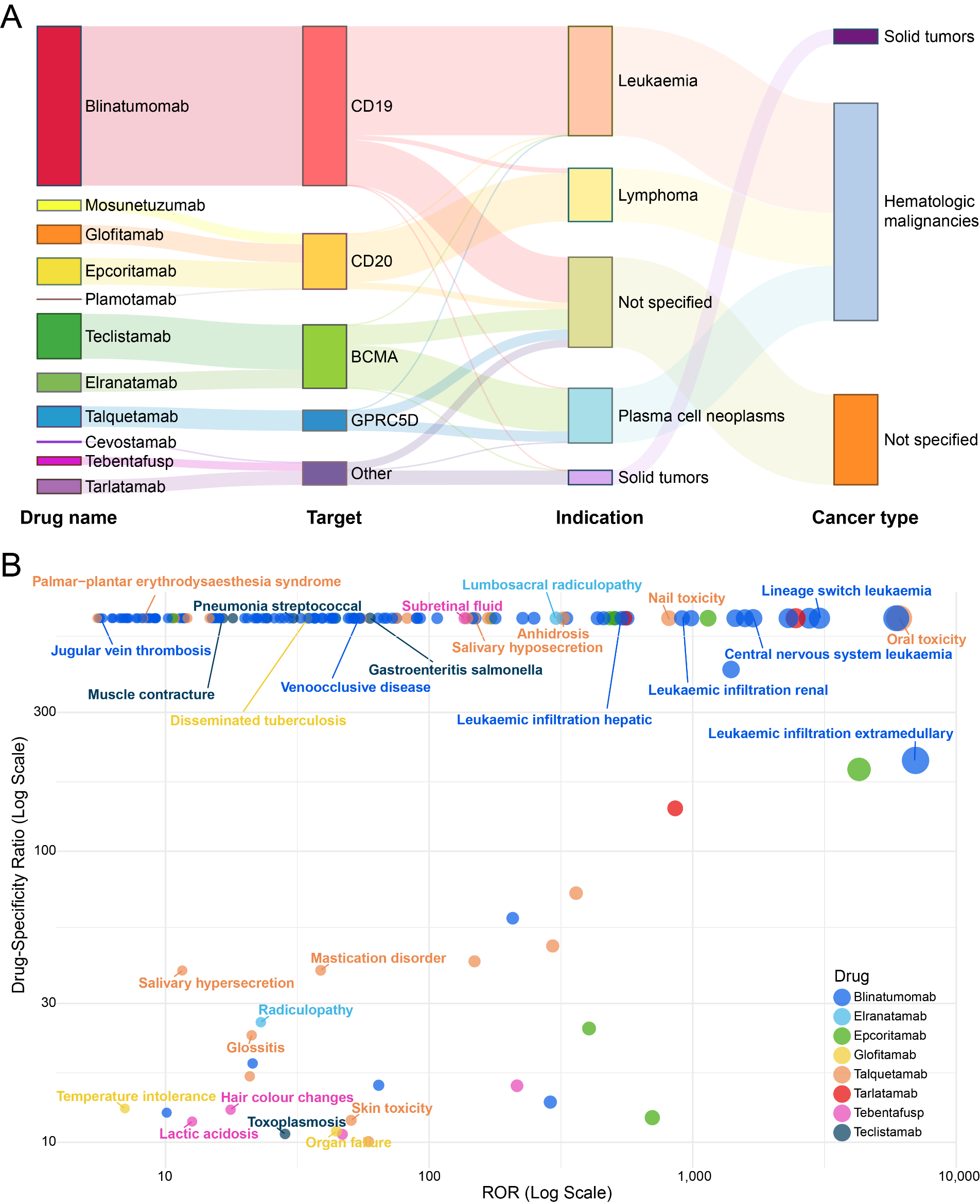


**Figure S2. T-cell Engager (TCE) Reporting Relationships and Unique Adverse Event Signals Identified in FAERS.**

(A) Sankey diagram illustrating the flow and relationships between individual TCE drug names, their molecular targets, reported clinical indications, and overarching cancer types, based on the FAERS database. The width of each flow is proportional to the number of reports. (B) Scatter plot identifying unique adverse event (AE) signals for specific TCE drugs. The x-axis represents the Reporting Odds Ratio (ROR) and the y-axis represents the Drug-Specificity Ratio (formerly Uniqueness Ratio), both on a log scale. A drug-AE pair was classified as a drug-specific signal if it met the hierarchical criteria: first, exhibiting standard statistical significance (lower bound of ROR 95% CI > 1 and case count a ≥ 3), and subsequently meeting prioritization thresholds of ROR > 5 and Drug-Specificity Ratio > 10. Many AEs located at the top of the plot have an infinite Drug-Specificity Ratio, indicating the event was reported for only a single drug in the cohort.


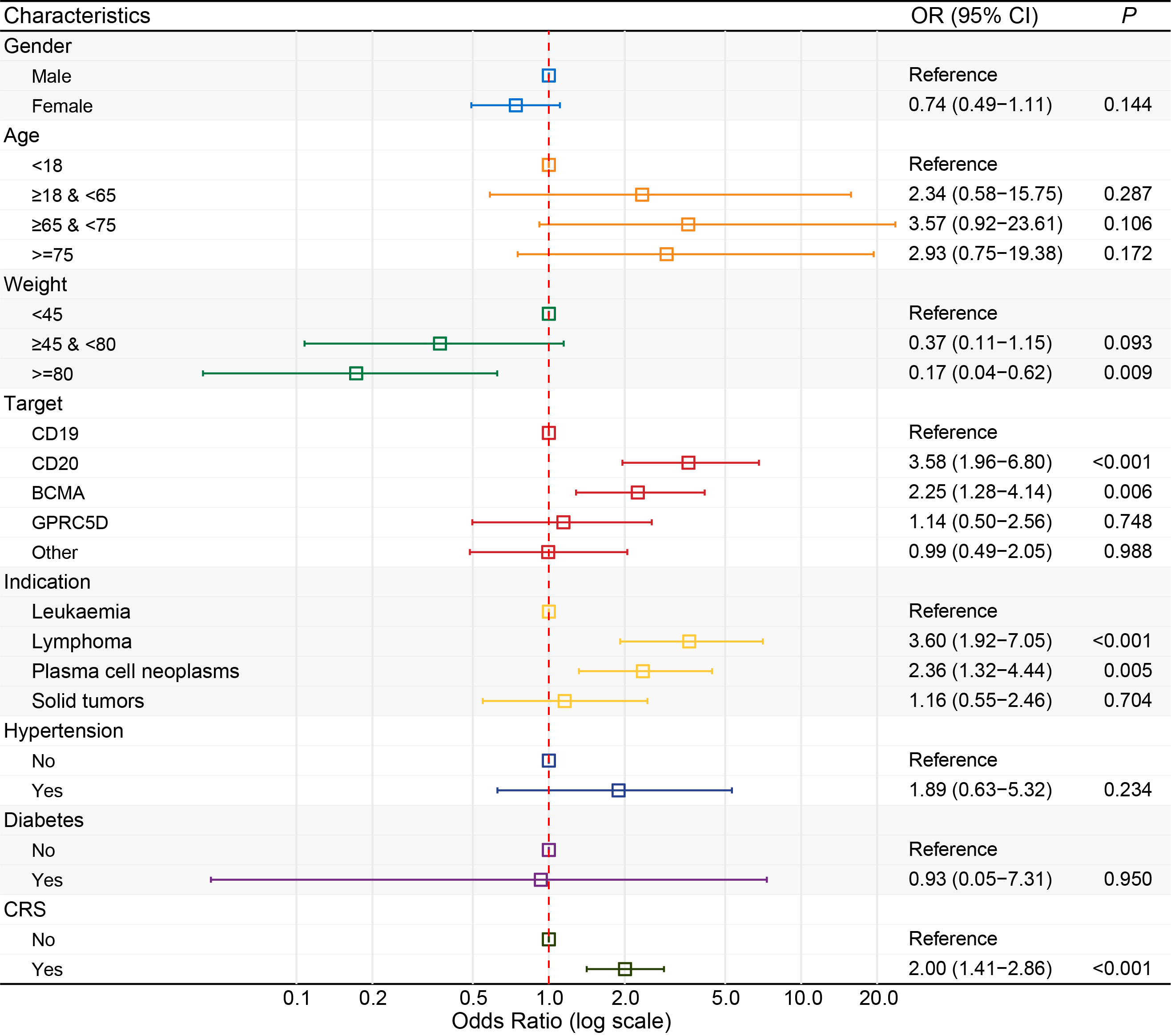


**Figure S3. Forest plot of Risk Factors for Fatal Outcomes in T-cell Engager (TCE)-Associated Immune Effector Cell-Associated Neurotoxicity Syndrome (ICANS) Based on FAERS Reports.**
