# Supplementary figures and images for: Real-world safety profile of T-cell engagers: evidence from multi-database analysis with CAR-T comparisons
Source: Front Immunol. 2026 Mar 6;17:1740144. doi: 10.3389/fimmu.2026.1740144 (PMC13002793; doi:10.3389/fimmu.2026.1740144)

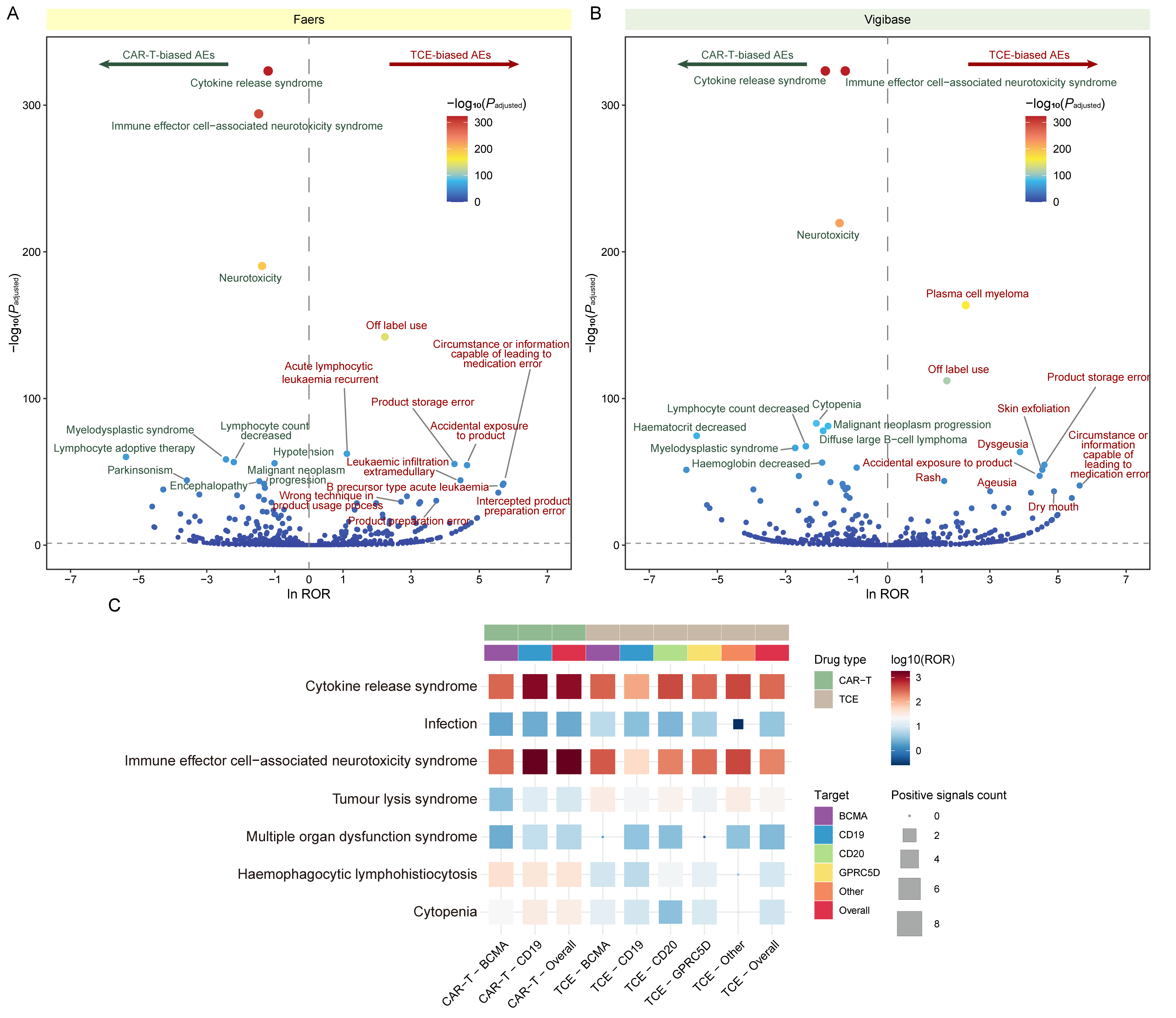

Supplement: Supplementary file 2 [file Image1.tif]

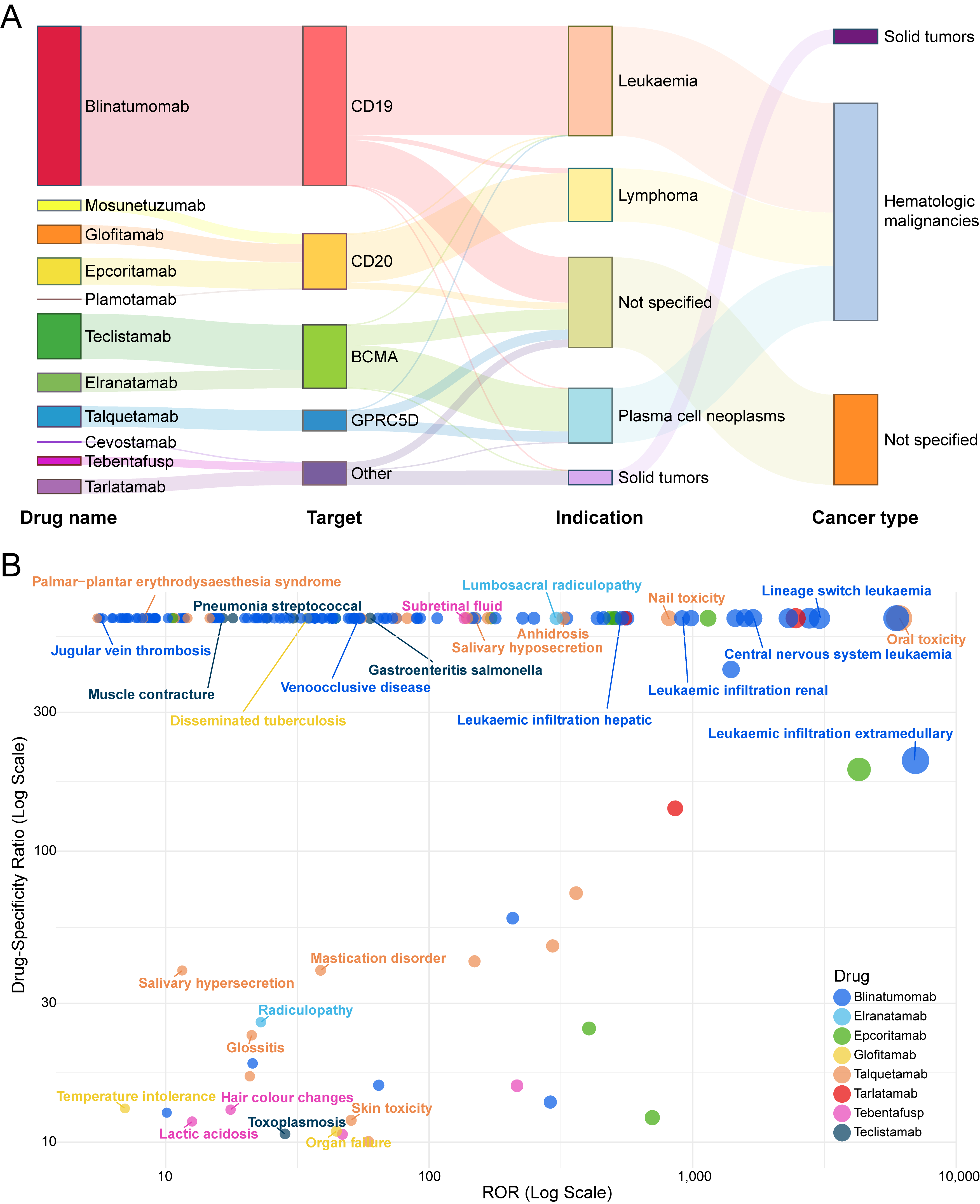

Supplement: Supplementary file 3 [file Image2.tif]

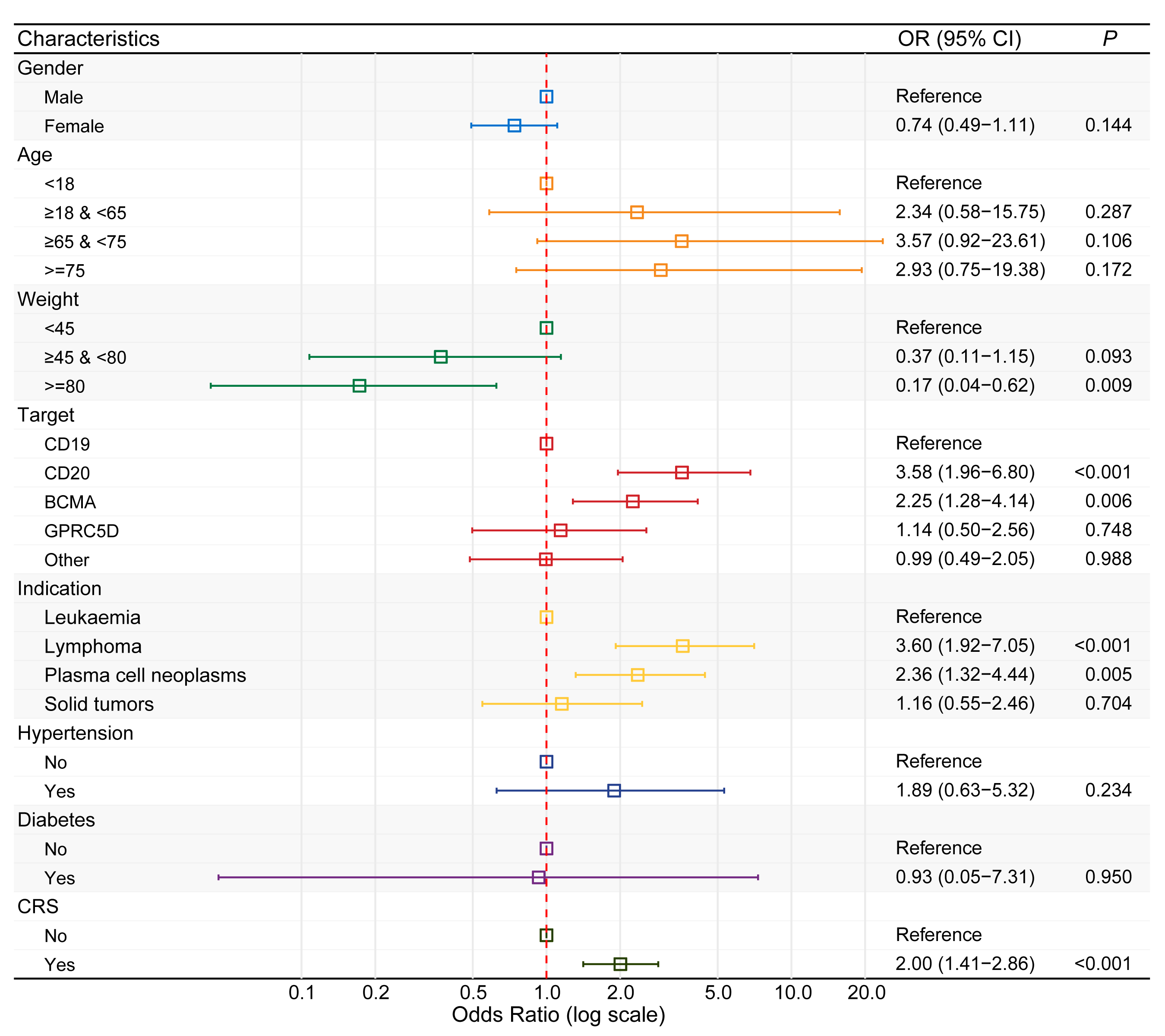

Supplement: Supplementary file 4 [file Image3.tif]
